# Comparative Evaluation of Serum Separator V-Tube™, VQ-Tube™, and K_2_EDTA V-Tube™ with Becton Dickinson Tubes for Chemistry, Immunology, and Hematology Examinations

**DOI:** 10.3390/diagnostics15141775

**Published:** 2025-07-14

**Authors:** Takho Kang, Seung Gyu Yun, Myung-Hyun Nam, Yunjung Cho, Minjeong Nam

**Affiliations:** Department of Laboratory Medicine, Korea University Anam Hospital, Korea University College of Medicine, Seoul 02841, Republic of Korea

**Keywords:** performance evaluation, vacuum blood collection tube, clinical significance

## Abstract

**Background:** Rigorous evaluation of vacuum blood collection tubes is essential to ensure the reliability of laboratory results. **Methods**: In this study, we compared the serum separator tube V-Tube™ (V-Tube SST), the quick-clotting serum separator tube VQ-Tube™ (VQ-Tube SST), and the K_2_EDTA V-Tube™ (V-Tube K_2_EDTA) manufactured by AB Medical (Seoul, Republic of Korea), with their respective counterparts from Becton Dickinson (BD, Franklin Lakes, NJ, USA): BD Vacutainer^®^ SST™ II Advance Tube (BD SST) and BD Vacutainer^®^ K_2_EDTA 5.4 mg Tube (BD K_2_EDTA). The evaluation encompassed 61 measurands across the fields of chemistry, immunology, and hematology, and incorporated a stability assessment for the VQ-Tube SST. **Results**: The V-Tube SST, VQ-Tube SST, and V-Tube K_2_EDTA demonstrated comparable analytical performance to the BD tubes for the majority of measurands. However, glucose, lactate dehydrogenase, mean corpuscular volume, and mean corpuscular hemoglobin concentration indicated clinically significant differences according to the desirable biases referenced from the Desirable Biological Variation Database specifications (Ricos) and the European Federation of Clinical Chemistry and Laboratory Medicine (EFLM). **Conclusions**: These findings suggest that, while the V-Tube SST, VQ-Tube, and V-Tube K_2_EDTA generally serve as alternatives to BD tubes, caution should be taken when interpreting results for specific measurands that demonstrated clinically significant discrepancies.

## 1. Introduction

The accuracy and reliability of laboratory test results are fundamentally dependent on minimizing pre-examination errors. Among the various pre-examination factors, the performance of vacuum blood collection tubes is particularly critical, as tube-related variables—including additive composition and serum-clotting time—can significantly influence the precision and stability of measurements [1,2].

Serum separator tubes (SSTs) and ethylenediaminetetraacetic acid (EDTA) tubes are widely used in clinical laboratories. SSTs, routinely used in clinical chemistry and immunology examinations, are designed with a clot activator and a gel barrier to promote the efficient separation of serum from cellular components following centrifugation [3]. Conventional SSTs require a 30 min clotting time and 10 min of centrifugation prior to analysis. However, quick-clotting SSTs utilize a thrombin-based clot activator that significantly reduces the clotting time to approximately 5 min [3], which is expected to enhance workflow efficiency without compromising analytical performance.

EDTA tubes, predominantly used for hematology testing, contain EDTA as an anticoagulant that chelates calcium and prevents coagulation. Both dipotassium (K_2_EDTA) and tripotassium (K_3_EDTA) formulations are widely used, depending on the specific analytical application and institutional preferences [4,5]. EDTA tubes are essential for preserving cellular morphology and preventing clot formation in whole blood samples analyzed for complete blood counts and related parameters.

Many studies have evaluated the performance of various blood collection tubes, including the serum separator tube V-Tube™ (V-Tube SST), the quick-clotting serum separator tube VQ-Tube™ (VQ-Tube SST), and the K_2_EDTA V-Tube™ (V-Tube K_2_EDTA), comparing them with established commercial products including the BD Vacutainer^®^ SST™ II Advance Tube (BD SST) and BD Vacutainer^®^ K_2_EDTA 5.4 mg Tube (BD K_2_EDTA tube). While these studies have demonstrated comparable analytical performance for the selected measurands, they have often assessed a limited number of measurands and used outdated evaluation criteria or insufficiently rigorous statistical methods [2,3,5,6,7,8,9,10,11].

This study aimed to comprehensively evaluate the measurement performance of V-Tube SST, VQ-Tube SST, and V-Tube K_2_EDTA in comparison with the corresponding BD SST and BD K_2_EDTA across a broad panel of chemistry, immunology, and hematology measurands. The clinical significance was assessed according to the desirable bias from the Desirable Biological Variation Database specifications (Ricos) and the European Federation of Clinical Chemistry and Laboratory Medicine (EFLM) [12,13]. In addition, this study assessed the measurement stability in samples collected using the VQ-Tube SST under defined storage conditions.

## 2. Materials and Methods

### 2.1. Sample Collection

This study was conducted at Korea University Anam Hospital (Seoul, Republic of Korea) between January and August 2023. A total of 40 medically eligible subjects, aged 19–69 years, with body weights exceeding 50 kg for males and 45 kg for females, were included in the study cohort. Subjects were excluded if they were pregnant, breastfeeding, had serious chronic conditions, or if their blood samples showed hemolysis, coagulation, lipemia, or icterus. For each subject, 26 mL of venous blood was collected using a Vacuette Multiple Use Needle (Greiner Bio-One, Kremsmünster, Austria) and distributed into six vacuum tubes: one 5.0 mL V-Tube SST (#321502; SST with barrier gel and clot activator; AB Medical, Seoul, Republic of Korea), two 5.0 mL VQ-Tube SST (#B2150B; SST with barrier gel and thrombin-based clot activator; AB Medical, Seoul, Republic of Korea), one 5.0 mL BD SST (#368640; SST with barrier gel and clot activator; Becton Dickinson, Franklin Lakes, NJ, USA), one 3.0 mL V-Tube K_2_EDTA (#510303; containing 5.1 mg K_2_EDTA; AB Medical, Seoul, Republic of Korea), and one 3.0 mL BD K_2_EDTA (#367859; containing 5.4 mg K_2_EDTA; Becton Dickinson, Franklin Lakes, NJ, USA). All tubes were filled to the full capacity of the collection tube, inverted ten times, and incubated in an upright position at room temperature for 30 min, except for one VQ-Tube SST sample, which was incubated for 5 min. Subsequently, all samples were centrifuged at 3500× *g* using a Kubota 5930 centrifuge (Kubota, Tokyo, Japan) and transferred to the laboratory automation system. Only samples without observable hemolysis, icterus, or lipemia were included, and all samples were processed without delay. This study was approved by the Institutional Review Board of Korea University (IRB No. 2023AN0135) and conducted in accordance with the Declaration of Helsinki. Written informed consent was obtained from all subjects. 

### 2.2. Assays

Chemistry examinations were performed using the Beckman Coulter AU5800 (Beckman Coulter, Brea, CA, USA), immunology examinations with the Roche Cobas 8000 (Roche Diagnostic, Rotkreuz, Switzerland) and Abbott Alinity i (Abbott Laboratories, Abbott Park, IL, USA), hematology examinations with the Sysmex XN-10 (Sysmex, Kobe, Japan), ESR with the TEST1 BCL (Alifax, Padova, Italy), and HbA1c with the Bio-Rad D-100 (Bio-Rad Laboratories, Hercules, CA, USA).

Comparative evaluations were conducted between the V-Tube SST and BD SST, and between the VQ-Tube SST and BD SST, for a total of 40 measurands. These included 29 chemistry examinations, such as aspartate aminotransferase (AST), alanine aminotransferase (ALT), γ-glutamyltransferase (GGT), alkaline phosphatase (ALP), total protein (TP), albumin (alb), total bilirubin (TB), direct bilirubin (DB), blood urea nitrogen (BUN), creatinine (Cr), total cholesterol (TC), low-density lipoprotein-cholesterol (LDL), high-density lipoprotein-cholesterol (HDL), triglyceride (TG), glucose, uric acid (UA), lactate dehydrogenase (LDH), creatine kinase (CK), amylase, lipase, total iron binding capacity (TIBC), sodium (Na), potassium (K), chloride (Cl), magnesium (Mg), calcium (Ca), inorganic phosphate (P), iron (Fe), C-reactive protein (CRP). Eleven immunology examinations were performed, including procalcitonin (PCT), α-fetoprotein (AFP), carcinoembryonic antigen (CEA), prostate-specific antigen (PSA), troponin-T, creatine kinase-MB (CK-MB), free thyroxine (fT4), thyroid-stimulating hormone (TSH), HBsAg, Anti-HCV, and HIV Ag/Ab combo. The stability of the VQ-Tube SST was evaluated by comparing the results of 29 chemical measurands after clotting times of 5 and 30 min.

V-Tube K_2_EDTA and BD K_2_EDTA were compared for 20 hematological examinations, including erythrocyte count (RBC), hemoglobin (Hb), hematocrit (Hct), mean corpuscular volume (MCV), mean corpuscular hemoglobin (MCH), mean corpuscular hemoglobin concentration (MCHC), red blood cell distribution width (RDW), reticulocyte count, immature reticulocyte fraction (IRF), white blood cell count (WBC), neutrophil count, lymphocyte count, monocyte count, eosinophil count, basophil count, platelet, plateletcrit, mean platelet volume (MPV), platelet distribution width (PDW), erythrocyte sedimentation rate (ESR), and hemoglobin A1c (HbA1c).

### 2.3. Statistical Analysis

Normality was assessed using the Shapiro–Wilk test. The error for a single paired measurement was defined as the difference between the candidate tube and reference tube result. For errors showing a parametric distribution, paired *t*-tests were performed; otherwise, the Wilcoxon signed-rank test was applied [1], with statistical significance set at *p* < 0.05. Bias, representing systematic difference, was calculated as the mean (or median) of errors, $\frac{1}{N}\sum_{i=1}^{N}candidate\;\left(i\right)-reference\;(i)$ , to minimize the imprecision by averaging [14,15]. Additionally, bias can be represented proportionally as the mean (or median) of proportional errors, $\frac{1}{N}\sum_{i=1}^{N}\frac{candidate\;\left(i\right)-reference\;(i)}{reference\;(i)}$ [15]. Bias was considered statistically significant if its value and 95% confidence interval (CI) did not include zero (line of equality) [14]. Difference plots visualize the bias between two methods, assuming a constant linear bias within a specified interval; the reference tube measurements were plotted on the horizontal axis [14,15]. Clinically significant differences were defined as cases in which the biases and their 95% CI exceeded the allowable limits of desirable biases [16], according to Ricos [12] and EFLM [13], as of July 2025.

Regression analyses were conducted to further characterize the biases [16,17]. Deming regression was used for normally distributed measurands; otherwise, Passing–Bablok regression was applied [1]. The absence of constant bias was inferred when the intercept’s 95% CI included zero, and the absence of proportional bias when the slope’s 95% CI included one [16,17]. All analyses were conducted using Analyze-it (version 6.15.4; Analyze-it Software, Leeds, UK).

## 3. Results

**Figure 1:**
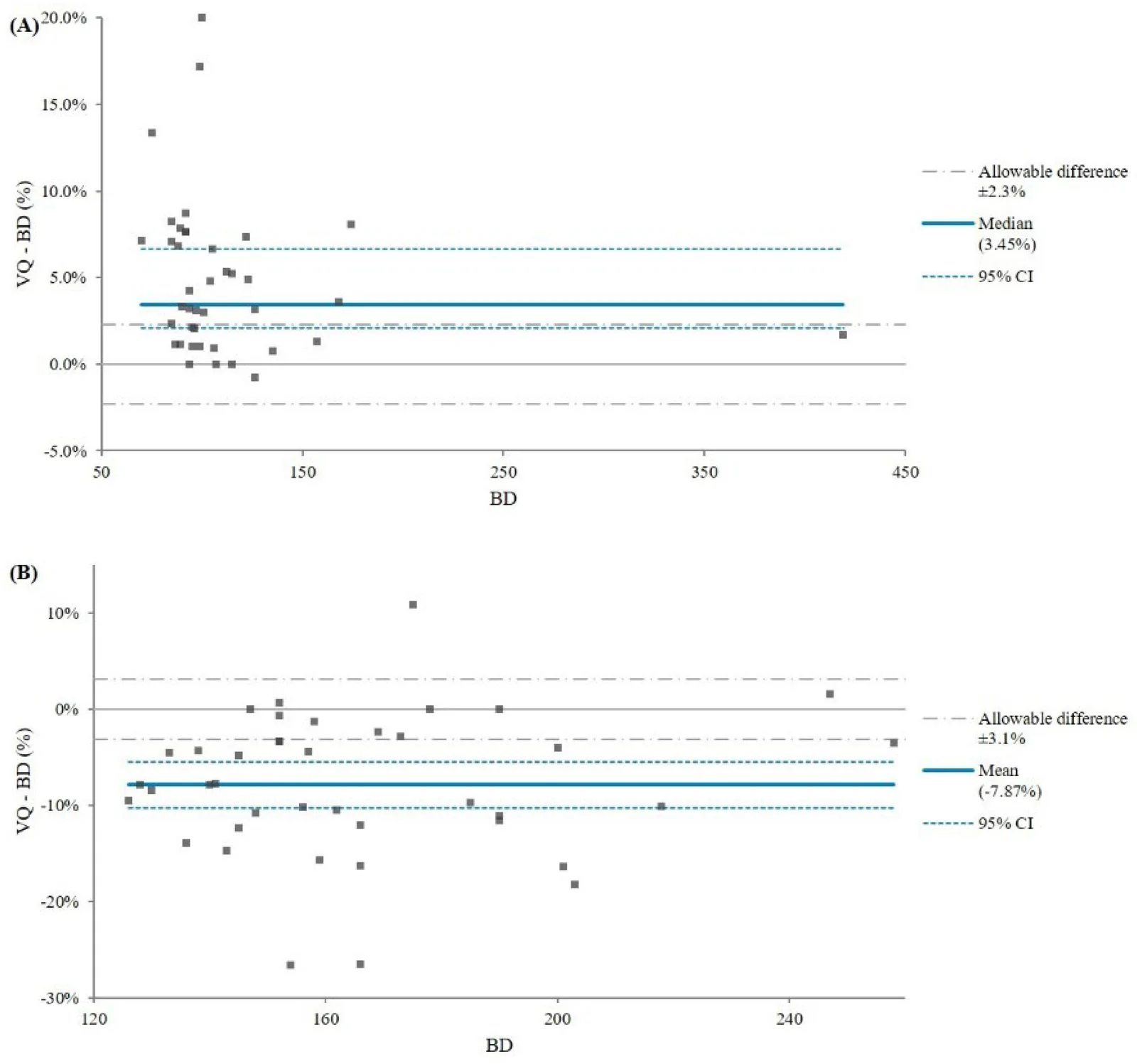
Difference plots for glucose and LDH comparing BD SST and VQ-tube SST. (**A**) Glucose and (**B**) LDH. For glucose, the bias and 95% CI (3.45%, 2.11–6.67%) overlapped with the EFLM desirable bias of 2.3%, but not with the Ricos desirable bias of 1.8%, and for LDH, the bias and 95% CI (−7.87%, −10.25–−5.49%) did not overlap with the EFLM desirable bias of 3.1%.

In the comparison of chemistry and immunology measurands between V-Tube SST and BD SST, statistically significant differences were observed for 14 measurands (Cr, HDL, UA, LDH, amylase, K, Ca, P, AFP, CK-MB, fT4, TSH, HBsAg, and HIV Ag/Ab). HDL, amylase, P, and AFP showed positive biases in the V-Tube SST, whereas Cr, UA, LDH, fT4, HBsAg, and HIV Ag/Ab showed negative biases compared to BD SST (Table 1). The biases (%) ranged from −8.86% to 2.49% between V-Tube SST and BD SST. The biases and 95% CIs for all measurands were within the allowable limits defined by Ricos and EFLM. In the comparison between VQ-Tube SST and BD SST, statistically significant differences were identified for 16 measurands (TD, TB, LDL, HDL, TG, glucose, UA, LDH, Na, Mg, P, AFP, CK-MB, fT4, TSH, and HBsAg). HDL, TG, glucose, and AFP showed positive biases in the VQ-Tube SST, whereas UA, LDH, fT4, and HBsAg showed negative biases. The biases ranged from −7.87% to 3.45% between VQ-Tube SST and BD SST. The biases and 95% CIs for all analytes were within the Ricos and EFLM allowable limits, except glucose and LDH. Glucose showed a clinically significant positive bias (3.45%; 95% CI, 2.11–6.67%), and LDH showed a clinically significant negative bias (−7.87%; 95% CI, −10.25–−5.49%) (Figure 1).

In the comparison between BD SST and V-Tube, PCT, HBsAg, and HIV Ag/Ab showed moderate to low correlations, with Spearman’s rho values of 0.667, 0.394, and 0.601, respectively (Table 2). In the comparison between BD SST and VQ-Tube SST, HBsAg indicated a moderate correlation with Spearman’s rho values of 0.526. All other measurands indicated high to very high correlations, with Spearman’s rho values greater than 0.7.

**Figure 2:**
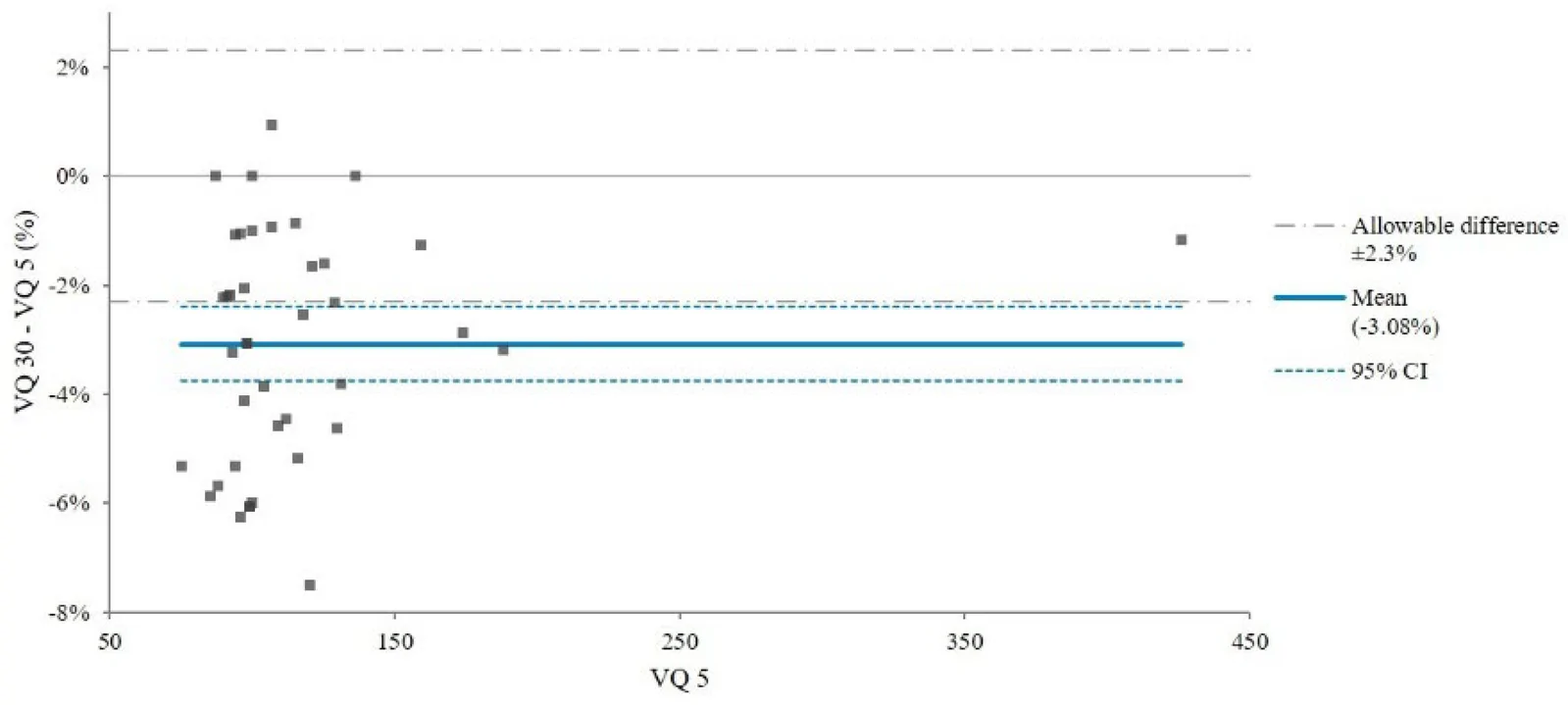
Difference plot for stability assessment of glucose between VQ-Tube SST with 5 min and 30 min clotting time. Bias and 95% CI (−3.08%, −3.76–−2.40%) did not overlap with the EFLM desirable bias of 2.3%.

In the stability test of VQ-Tube SST, statistically significant differences were observed in GGT, glucose, UA, LDH, K, Ca, and Fe (Table 3). LDH showed positive biases in the VQ-Tube SST with 30 min clotting time, whereas glucose, UA, and CK showed negative biases. The biases (%) ranged from −3.08% to 4.60%. For almost all measurands, the biases and 95% CIs were within allowable limits, although glucose exceeded the Ricos and EFLM. Glucose showed a clinically significant negative bias (−3.08%; 95% CI, −3.76–−2.40%) (Figure 2). All measurands showed high to very high correlation between VQ-Tube SST with 5 min and 30 min clotting times.

**Figure 3:**
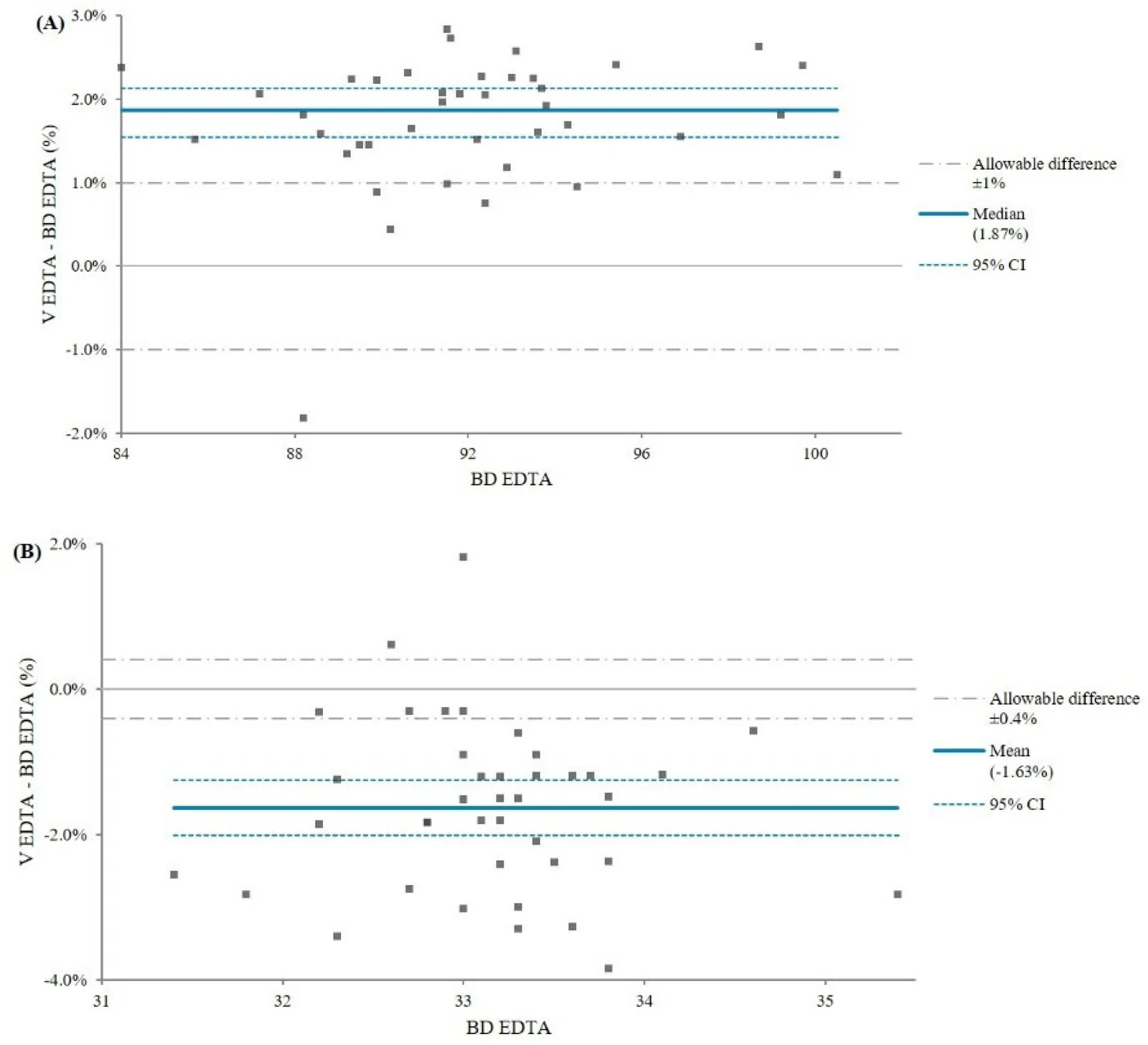
Difference plots comparing V-Tube K_2_EDTA and BD K_2_EDTA for: (**A**) MCV, (**B**) MCHC. For MCV, the bias and 95% CI (1.87%, 1.55–2.13%) did not overlap with the EFLM desirable bias of 1.0%, and for MCHC, the bias and 95% CI (−1.63%, −2.01–−1.26%) did not overlap with the EFLM desirable bias of 0.4%.

Among hematological measurands, Hct, MCV, MCHC, RDW, and ESR showed statistically significant differences between V-Tube K_2_EDTA and BD K_2_EDTA tubes. Hct, MCV, IRF, and eosinophil showed positive biases in the V-Tube K_2_EDTA, whereas MCHC showed negative biases. (Table 4). The biases (%) ranged from −5.71% to 6.83%. The biases and 95% CIs for all measurands were within the Ricos and EFLM, with the exception of MCV and MCHC. MCV showed a clinically significant positive bias (1.87%; 95% CI, 1.55–2.13%), whereas MCHC showed a clinically significant negative bias (−1.63%; 95% CI, −2.01–−1.26%) (Figure 3).

## 4. Discussion

Vacuum blood collection tubes are indispensable in clinical medicine, as a large portion of medical decision-making relies on blood test results. However, the accuracy of these measurements can be influenced by various factors, including clotting time and the composition of additives [1].

A comparison between the V-Tube SST and the BD SST demonstrated that all measurands for chemistry and immunology satisfied Ricos and EFLM. Therefore, these findings support the clinical interchangeabilities of the V-Tube SST and the BD SST within the evaluated measurement intervals for the tested measurands. Although the constant and proportional biases were observed for cholesterol, HDL, and LDH, these biases did not result in clinically significant overall biases; nevertheless, further studies would be needed. These findings are consistent with previous studies, which also identified statistically significant differences for certain measurands but without clinically significant differences [6,7].

In contrast, when the VQ-Tube SST was compared to the BD SST, glucose and LD demonstrated clinically significant differences according to Ricos or EFLM. The VQ-Tube SST, designed for rapid clotting, requires a clotting time of 5 min, compared to the conventional SST’s 30 min clotting times. The shortened clotting and reduced serum–clot contact times have been shown to limit glycolytic activity, thereby preserving glucose concentrations, while simultaneously minimizing cellular lysis and the consequent release of LDH into the serum [2,3,10,11]. A previous study also indicated that the VQ-Tube SST is not interchangeable with the BD SST for glucose and LDH measurements [2].

Stability testing of the VQ-Tube SST revealed a clinically significant decrease in glucose concentrations with prolonged serum–clot contact time, attributable to ongoing glycolysis. In contrast, LDH did not show a substantial difference, likely because serum LDH levels, while known to increase with contact time [2,3,10,11], do not rise significantly until after 4 h [18]. The contribution of the Fe constant bias to the overall bias was insignificant.

Regarding K_2_EDTA tubes, the V-Tube K_2_EDTA contains 5.1 mg of K_2_EDTA (5.82 mmol/L in a 3.0 mL tube), while the BD K_2_EDTA contains 5.4 mg of K_2_EDTA (6.16 mmol/L in a 3.0 mL tube). Both comply with ISO 6710 standards, specifying an acceptable EDTA concentration range of 4.11–6.843 mmol/L [19,20]. However, higher EDTA concentrations increase plasma tonicity, leading to erythrocyte shrinkage and reduced Hct [20,21]. While the bias of the Hct is not clinically significant, its derivative parameters, including MCV ( $\frac{Hct}{RBC}$ ) and MCHC ( $\frac{MCH}{MCV}$ ), exhibit clinically significant differences due to the magnification of discrepancy in their calculations. For ESR, desirable bias is unavailable from Ricos and EFLM [12,13,22]. A previous study has referenced a ±10% total allowable error, as recommended by the manufacturer Alifax (Padova, Italy) [5]. In this study, an allowable bias of 5%, corresponding to half of the total allowable error, was arbitrarily selected. Although ESR bias were within this predefined range, regression analysis indicated a significant proportional bias; however, its pattern of ESR increasing rather than decreasing at higher EDTA concentrations is contrary to theoretical expectations. Microcytic erythrocytes resulting from elevated EDTA concentrations exhibit an increased surface-to-volume ratio and a relatively higher negative surface charge density, both of which would be expected to decrease ESR [23,24]. Additionally, higher EDTA concentrations are anticipated to chelate more Ca^2+^, which would also be expected to reduce ESR. However, contrary to these expectations, the present study observed higher ESR at increased EDTA concentrations, suggesting that additional factors may be influencing. A previous study has similarly reported clinically significant differences in MCV and MCHC between these tubes, supporting the recommendation to use a single brand of tube for these measurements [9]. In summary, the V-Tube K_2_EDTA may not be fully interchangeable with the BD K_2_EDTA for the measurement of MCV and MCHC according to the Ricos and EFLM. Further evaluation is warranted to determine the comparability of ESR results between the V-Tube K_2_EDTA and the BD K_2_EDTA.

Although correlation is commonly used for method comparison, it has inherent limitations. It primarily reflects the strength of the linear relationship between two methods and does not necessarily indicate agreement or comparability [17]. Furthermore, a high correlation coefficient does not guarantee the absence of bias [17,25]. Additionally, the restricted range of the measurands also lowers the correlation coefficient [25]. In our study, many measurands with lower correlation coefficients were associated with a narrow distribution range.

This study has several limitations. Although the inclusion of 40 subjects meets the minimum sample size recommended by CLSI EP09C-ED3 [15], a larger cohort would enhance the precision of the measurands and mitigate the restricted range effect. In addition, evaluations involving subjects with diverse underlying conditions are warranted, as factors such as anticoagulant use, hormone replacement therapy, and various medications may affect measurement outcomes [3,26,27]. Moreover, the specification for allowable bias is not a fixed criterion; it is periodically updated, influenced by the population specificity of the underlying database. It should be selected in accordance with the intended clinical application [28,29]. Therefore, future studies involving larger and more clinically diverse subjects are warranted to enhance the generalizability of the findings. In addition, careful selection of updated and context-specific performance criteria is essential to ensure robust validation and broader applicability of alternative vacuum blood collection tubes in clinical practice.

## 5. Conclusions

This study demonstrated that the V-Tube SST, VQ-Tube SST, and V-Tube K_2_EDTA are clinically interchangeable with the BD SST and BD K_2_EDTA for routine chemistry, immunology, and hematology examinations. However, the VQ-Tube SST requires careful consideration when used for glucose and LDH measurements, given the observed clinically significant differences. Although the EDTA concentration in the V-Tube K_2_EDTA complies with established standards, its influence on MCV and MCHC highlights the potential impact of tube composition on hematological parameters. These findings underscore the necessity of tube-specific validation prior to clinical implementation. Further studies involving larger and more diverse subjects are needed to ensure reliable and consistent laboratory results across different blood collection systems.

## Figures and Tables

**Table 1:** Comparative analysis of chemistry and immunology measurands between BD SST, V-Tube SST, and VQ-tube SST.

Measurand	BD SST (Mean/Median)	V-Tube SST			VQ-Tube SST			Ricos (%)	EFLM (%)
		Mean/Median	*p*	Bias (%) (95% CI)	Mean/Median	*p*	Bias (%) (95% CI)		
AST, U/L	21.5 (18.0–25.6)	21.5 (18.0–25.0)	0.34	0.00 (−2.86–0.00)	21.5 (18.0–25.6)	0.34	0.00 (0.00–0.00)	6.54	5.3
ALT, U/L	14.0 (12.4–21.6)	15.0 (12.0–21.0)	0.79	0.00 (0.00–0.00)	14.0 (11.4–21.0)	0.37	0.00 (0.00–0.00)	11.48	8.1
GGT, U/L	13.0 (11.0–30.5)	13.0 (11.0–30.1)	0.21	0.00 (0.00–0.00)	13.5 (12.0–30.1)	1.00	0.00 (0.00–0.00)	11.06	11.5
ALP, U/L	54.5 (47.0–81.0)	55.0 (46.4–80.0)	0.25	−0.26 (−0.82–0.29) *	55.0 (47.0–82.0)	0.64	0.00 (0.00–0.00)	6.72	5.5
TP, g/dL	7.1 (6.8–7.4)	7.1 (6.9–7.4)	0.11	0.00 (0.00–1.30)	7.2 (6.9–7.4)	0.04	0.00 (0.00–1.43)	1.36	1.3
Alb, g/dL	4.5 (4.4–4.7)	4.5 (4.4–4.6)	0.85	0.00 (0.00–0.00)	4.5 (4.4–4.7)	0.66	0.00 (0.00–0.00)	1.43	1.2
TB, mg/dL	0.61 ± 0.18 *	0.61 (0.48–0.68)	0.23	0.00 (0.00–1.70)	0.62 (0.47–0.69)	0.03	1.56 (0.00–1.85)	8.95	8.0
DB, mg/dL	0.12 ± 0.04 *	0.12 ± 0.04 *	0.90 *	0.00 (0.00–5.56)	0.12 ± 0.04 *	0.69	0.00 (0.00–0.00)	14.2	N/A
BUN, mg/dL	12.8 (10.3–16.6)	12.9 (10.4–16.6)	0.21 *	0.24 (−0.16–0.64) *	12.8 (10.4–16.6)	0.48	0.00 (0.00–0.65)	5.57	6.0
Cr, mg/dL	0.75 (0.66–0.83)	0.75 (0.66–0.83)	0.02 *	−1.01 (−1.79–−0.22) *	0.75 (0.66–0.82)	0.39	−0.18 (−0.86–0.50) *	3.96	4.2
Cholesterol, mg/dL	187.3 ± 34.6 *	187.5 ± 35.7 *	0.92	0.02 (−0.38–0.43) *	187.9 ± 35.3 *	0.34	0.28 (−0.16–0.73) *	4.1	4.1
LDL, mg/dL	105.8 ± 31.2 *	106.1 ± 31.2 *	0.14	0.00 (0.00–1.01)	106.6 ± 31.3 *	0.009	0.00 (0.00–0.99)	5.46	5.6
HDL, mg/dL	61.9 ± 13.0 *	63.3 ± 12.6 *	<0.0001 *	2.49 (1.52–3.46) *	63.4 ± 12.8 *	<0.0001 *	2.62 (1.64–3.61) *	5.61	5.5
TG, mg/dL	101.0 (61.8–157.6)	102.0 (63.0–157.2)	0.22	0.00 (0.00–0.88)	102.0 (62.8–158.8)	<0.0001 *	1.07 (0.54–1.60) *	9.57	9.9
Glucose, mg/dL	98.0 (90.8–115.0)	99.5 (90.4–114.9)	0.14	0.00 (0.00–1.05)	100.0 (94.8–120.6)	<0.0001 *	3.45 (2.11–6.67)	1.8	2.3
UA, mg/dL	4.63 (4.03–5.32)	4.61 (4.01–5.29)	0.0003 *	−0.56 (−0.85–−0.27) *	4.60 (3.99–5.29)	0.03 *	−0.33 (−0.61–−0.05) *	4.87	N/A
LDH, U/L	157.5 (145.0–182.1)	148.0 (134.4–171.6)	0.0002	−4.68 (−6.96–−2.41) *	147.0 (129.4–168.0)	<0.0001 *	−7.87 (−10.25–−5.49) *	4.3	3.1
CK, U/L	101.0 (76.0–151.3)	98.0 (75.0–150.3)	0.67 *	−0.14 (−1.00–0.72) *	99.5 (76.0–151.7)	0.70	0.00 (−0.54–1.02)	11.5	8.9
Amylase, U/L	60.0 (44.8–71.0)	61.0 (45.3–70.6)	0.03	0.74 (0.04–1.43) *	61.0 (44.8–71.2)	0.24	0.41 (−0.35–1.17) *	7.4	7.5
Lipase, U/L	26.0 (15.0–31.6)	25.5 (15.0–32.0)	1.00	0.00 (0.00–0.00)	25.5 (15.4–33.0)	0.41	0.00 (0.00–0.00)	11.31	6.3
TIBC, μg/dL	337.1 ± 50.7 *	337.5 ± 51.3 *	0.78	0.00 (−0.34–0.32)	337.9 ± 51.5 *	0.22 *	0.24 (−0.17–0.65) *	N/A	N/A
Na, mmol/L	139.3 ± 2.1 *	140.0 (138.0–141.0)	0.61	0.00 (0.00–0.00)	138.9 ± 2.2 *	0.009	−0.35 (−0.71–0.00)	0.23	0.2
K, mmol/L	4.1 (3.9–4.4)	4.2 (3.9–4.4)	0.003	2.44 (0.00–2.50)	4.0 (3.9–4.3)	0.07	0.00 (−2.27–0.00)	1.81	1.7
Cl, mmol/L	104.0 (103.0–105.0)	104.0 (103.0–105.0)	0.60	0.00 (0.00–0.00)	104.0 (103.0–105.0)	0.87	0.00 (0.00–0.00)	0.5	0.4
Mg, mg/dL	0.8 (0.8–0.9)	0.9 (0.8–0.9)	0.13	0.00 (0.00–0.00)	0.9 (0.8–0.9)	0.008	0.00 (0.00–0.00)	1.8	1.6
Ca, mg/dL	9.7 (9.4–9.9)	9.7 (9.5–9.9)	0.02	0.50 (0.00–1.04)	9.7 (9.4–9.9)	0.43	0.00 (0.00–1.02)	0.82	0.7
P, mg/dL	3.85 ± 0.46 *	3.86 ± 0.46 *	0.04 *	0.41 (0.03–0.80) *	3.87 ± 0.44 *	0.02 *	0.56 (−0.24–1.12)	3.38	3.3
Fe, µg/dL	91.1 ± 36.1 *	90.8 ± 36.0 *	0.21	0.00 (−0.74–0.00)	90.9 ± 36.3 *	0.47 *	0.00 (−0.81–0.00)	8.8	9.6
CRP, mg/L	0.60 (0.26–1.03)	0.58 (0.25–1.01)	0.37	0.00 (−1.49–0.00)	0.60 (0.26–1.01)	0.23	0.00 (0.00–2.61)	21.8	23.4
PCT, ng/mL	0.03 (0.02–0.04)	0.03 (0.02–0.04)	0.80	0.00 (0.00–0.00)	0.03 (0.02–0.03)	0.30	0.00 (0.00–0.00)	N/A	N/A
AFP, ng/mL	2.66 (1.86–3.52)	2.70 (1.83–3.54)	0.02	1.31 (0.17–2.45) *	2.71 (1.87–3.50)	0.004 *	2.03 (0.84–2.86)	11.8	13.8
CEA, ng/mL	1.42 (1.00–2.07)	1.41 (0.98–2.06)	0.19	−0.45 (−1.24–0.34) *	1.45 (0.97–2.09)	0.80 *	0.00 (−1.12–1.49)	14.3	14.9
PSA, ng/mL	0.01 (0.01–0.18)	0.01 (0.01–0.20)	0.63	0.00 (0.00–0.00)	0.01 (0.01–0.20)	0.84	0.00 (0.00–0.00)	18.7	10.6
Troponin-T, ng/mL	0.003 (0.003–0.005)	0.003 (0.003–0.005)	0.25	0.00 (0.00–0.00)	0.003 (0.003–0.004)	0.31	0.00 (0.00–0.00)	23.7	8.2
CK-MB, ng/mL	1.32 (1.01–1.86)	1.31 (1.03–1.87)	0.01	0.82 (−0.45–2.27)	1.33 (1.04–1.86)	0.02 *	0.93 (0.00–2.51)	14.88	N/A
fT4, ng/dL	0.94 ± 0.11 *	0.93 ± 0.10 *	0.002 *	−1.58 (−2.67–−0.49) *	0.91 (0.83–1.02)	0.002 *	−1.94 (−3.14–−0.74) *	3.3	2.3
TSH, μIU/mL	1.78 ± 0.91 *	1.76 ± 0.91 *	0.04	−0.63 (−1.55–0.00)	1.76 ± 0.89 *	0.03	0.00 (−1.14–0.40)	7.8	10.0
HBsAg, S/CO	0.33 (0.29–0.35)	0.28 (0.26–0.32)	0.001	−8.86 (−12.93–−4.80) *	0.30 (0.27–0.32)	0.005	−6.74 (−10.55–−2.93) *	N/A	N/A
Anti-HCV, S/CO	0.06 (0.05–0.07)	0.06 (0.04–0.07)	0.27	0.00 (0.00–0.00)	0.05 (0.04–0.07)	0.08	0.00 (0.00–0.00)	N/A	N/A
HIV Ag/Ab, S/CO	0.07 (0.07–0.09)	0.07 (0.06–0.09)	0.008	−6.80 (−12.92–−0.68) *	0.07 (0.06–0.08)	0.08	0.00 (−11.11–0.00)	N/A	N/A

* Parametric analyses (mean ± SD, paired *t*-test, Deming regression, and Pearson correlation) were applied; otherwise, nonparametric analyses (median [1st and 3rd quartile], Wilcoxon signed rank test, Passing–Bablok regression, and Spearman’s correlation) were applied. Abbreviations: SST, serum separator tube; CI, confidence interval; AST, aspartate aminotransferase; ALT, alanine aminotransferase; GGT, gamma-glutamyl transferase; ALP, alkaline phosphatase; TP, total protein; Alb, albumin; TB, total bilirubin; DB, direct bilirubin; BUN, blood urea nitrogen; Cr, creatinine; Cholesterol, total cholesterol; LDL, low-density lipoprotein cholesterol; HDL, high-density lipoprotein cholesterol; TG, triglycerides; UA, uric acid; LDH, lactate dehydrogenase; CK, creatine kinase; TIBC, total iron-binding capacity; Na, sodium; K, potassium; Cl, chloride; Mg, magnesium; Ca, calcium; P, phosphorus; Fe, iron; CRP, C-reactive protein; PCT, procalcitonin; AFP, alpha-fetoprotein; CEA, carcinoembryonic antigen; PSA, prostate-specific antigen; CK-MB, creatine kinase-MB; fT4, free thyroxine; TSH, thyroid-stimulating hormone; HBsAg, hepatitis B surface antigen; Anti-HCV, hepatitis C virus antibody; HIV Ag/Ab, human immunodeficiency virus antigen/antibody.

**Table 2:** Correlation analysis between BD SST, V-Tube SST, and VQ-tube SST for chemistry and immunology measurands.

Measurand	BD and V-Tube SST			BD and VQ-Tube SST		
	Intercept (95% CI)	Slope (95% CI)	ρ (95% CI)	Intercept (95% CI)	Slope (95% CI)	ρ (95% CI)
AST, U/L	0.00 (−0.50–1.83)	1.00 (0.90–1.00)	0.982 (0.966–0.991)	0.00 (0.00–1.50)	1.00 (0.92–1.00)	0.986 (0.974–0.993)
ALT, U/L	0.00 (0.00–0.76)	1.00 (0.96–1.00)	0.983 (0.968–0.991)	0.00 (0.00–0.00)	1.00 (1.00–1.00)	0.995 (0.990–0.997)
GGT, U/L	0.00 (0.00–0.00)	1.00 (1.00–1.00)	0.986 (0.973–0.993)	0.00 (0.00–0.00)	1.00 (1.00–1.00)	0.986 (0.974–0.993)
ALP, U/L	0.00 (−1.00–0.83)	1.00 (0.98–1.00)	0.997 (0.995–0.999)	0.00 (−1.71–0.00)	1.00 (1.00–1.02)	0.997 (0.994–0.998)
TP, g/dL	0.00 (0.00–1.00)	1.00 (1.00–1.00)	0.976 (0.954–0.988)	0.00 (0.00–1.06)	1.00 (0.86–1.00)	0.967 (0.936–0.983)
Alb, g/dL	0.00 (0.00–0.00)	1.00 (1.00–1.00)	0.908 (0.828–0.951)	0.00 (0.00–0.00)	1.00 (1.00–1.00)	0.912 (0.836–0.954)
TB, mg/dL	0.00 (−0.01–0.02)	1.00 (0.97–1.03)	0.988 (0.977–0.994)	0.01 (−0.02–0.02)	1.00 (0.98–1.05)	0.988 (0.977–0.994)
DB, mg/dL	0.00 (−0.02–0.02) *	1.00 (0.86–1.14) *	0.936 (0.881–0.966) *	0.00 (−0.02–0.01) *	1.04 (0.89–1.18) *	0.953 (0.913–0.975) *
BUN, mg/dL	0.05 (−0.12–0.10)	1.00 (1.00–1.01)	0.997 (0.995–0.999)	0.00 (−0.21–0.22)	1.00 (0.98–1.02)	0.997 (0.994–0.998)
Cr, mg/dL	−0.02 (−0.05–0.00)	1.02 (1.00–1.06)	0.991 (0.982–0.995)	0.00 (−0.02–0.01)	1.00 (1.00–1.03)	0.988 (0.978–0.994)
Cholesterol, mg/dL	−5.86 (−10.62–−1.09) *	1.03 (1.01–1.06) *	0.998 (0.996–0.999) *	−3.53 (−8.24–1.17) *	1.02 (1.00–1.05) *	0.997 (0.995–0.999) *
LDL, mg/dL	0.41 (−1.53–2.34) *	1.00 (0.98–1.02) *	0.999 (0.998–0.999) *	0.44 (−1.54–2.43) *	1.00 (0.98–1.02) *	0.998 (0.997–0.999) *
HDL, mg/dL	2.95 (0.41–5.50) *	0.97 (0.94–1.01) *	0.992 (0.984–0.996) *	2.07 (−0.77–4.90) *	0.99 (0.94–1.04) *	0.990 (0.982–0.995) *
TG, mg/dL	0.00 (−0.54–1.00)	1.00 (1.00–1.01)	0.999 (0.999–1.000)	0.61 (−0.46–1.11)	1.01 (1.00–1.02)	0.999 (0.999–1.000)
Glucose, mg/dL	0.00 (−4.12–1.00)	1.00 (1.00–1.04)	0.993 (0.986–0.996)	4.50 (−2.40–9.17)	1.00 (0.96–1.07)	0.960 (0.923–0.979)
UA, mg/dL	−0.02 (−0.06–0.03)	1.00 (0.99–1.01)	0.993 (0.986–0.996)	−0.01 (−0.06–0.04)	1.00 (0.99–1.01)	0.995 (0.990–0.997)
LDH, U/L	−23.71 (−42.42–−8.00)	1.10 (1.00–1.21)	0.894 (0.803–0.944)	−20.58 (−45.50–−0.50)	1.07 (0.91–1.21)	0.858 (0.741–0.924)
CK, U/L	0.00 (−2.69–2.66)	1.00 (0.97–1.02)	0.996 (0.992–0.998)	0.00 (−2.31–2.08)	1.00 (0.99–1.02)	0.993 (0.987–0.996)
Amylase, U/L	0.00 (0.00–1.00)	1.00 (1.00–1.00)	0.997 (0.994–0.998)	0.00 (−1.02–1.00)	1.00 (1.00–1.02)	0.997 (0.994–0.998)
Lipase, U/L	0.00 (−1.08–0.00)	1.00 (1.00–1.04)	0.995 (0.990–0.997)	0.00 (0.00–0.71)	1.00 (0.97–1.00)	0.993 (0.986–0.996)
TIBC, μg/dL	−3.10 (−10.89–4.70) *	1.01 (0.99–1.03) *	0.998 (0.996–0.999) *	−4.02 (−12.68–4.65) *	1.01 (0.99–1.04) *	0.997 (0.994–0.998) *
Na, mmol/L	0.00 (−46.67–0.00)	1.00 (1.00–1.33)	0.954 (0.913–0.976)	−6.29 (−25.09–12.52) *	1.04 (0.91–1.18) *	0.943 (0.893–0.969) *
K, mmol/L	0.10 (−0.72–0.60)	1.00 (0.88–1.20)	0.933 (0.874–0.965)	0.00 (−0.05–1.01)	1.00 (0.75–1.00)	0.852 (0.732–0.921)
Cl, mmol/L	0.00 (0.00–0.00)	1.00 (1.00–1.00)	0.887 (0.792–0.940)	0.00 (0.00–0.00)	1.00 (1.00–1.00)	0.930 (0.868–0.963)
Mg, mg/dL	0.00 (0.00–0.00)	1.00 (1.00–1.00)	0.864 (0.753–0.928)	0.00 (0.00–0.00)	1.00 (1.00–1.00)	0.788 (0.626–0.885)
Ca, mg/dL	0.05 (0.00–0.10)	1.00 (1.00–1.00)	0.941 (0.889–0.969)	0.00 (−1.44–0.10)	1.00 (1.00–1.14)	0.927 (0.863–0.962)
P, mg/dL	0.00 (−0.10–0.10) *	1.00 (0.98–1.03) *	0.995 (0.990–0.997) *	0.16 (−0.09–0.41) *	0.96 (0.90–1.03) *	0.992 (0.984–0.996) *
Fe, µg/dL	0.15 (−0.62–0.93) *	1.00 (0.99–1.00) *	1.00 (0.999–1.000) *	−0.72 (−1.72–0.28) *	1.01 (0.99–1.02) *	0.999 (0.998–1.000) *
CRP, mg/L	0.00 (−0.01–0.01)	0.99 (0.98–1.00)	0.997 (0.994–0.998)	0.00 (0.00–0.01)	1.00 (0.99–1.02)	0.996 (0.992–0.998)
PCT, ng/mL	0.00 (0.00–0.00)	1.00 (1.00–1.00)	0.667 (0.441–0.814)	0.00 (0.00–0.00)	1.00 (1.00–1.00)	0.748 (0.562–0.862)
AFP, ng/mL	0.00 (−0.06–0.06)	1.01 (0.99–1.04)	0.993 (0.987–0.997)	0.02 (−0.03–0.08)	1.01 (0.98–1.03)	0.992 (0.985–0.996)
CEA, ng/mL	−0.01 (−0.04–0.02)	1.00 (0.98–1.02)	0.998 (0.996–0.999)	−0.01 (−0.05–0.01)	1.01 (0.99–1.03)	0.998 (0.995–0.999)
PSA, ng/mL	0.00 (0.00–0.00)	1.00 (1.00–1.03)	1.000 (1.000–1.000)	0.00 (0.00–0.00)	1.00 (1.00–1.00)	1.000 (1.000–1.000)
Troponin-T, ng/mL	0.00 (0.00–0.00)	1.00 (1.00–1.03)	1.000 (0.999–1.000)	0.00 (0.00–0.00)	1.00 (1.00–1.00)	0.883 (0.785–0.938)
CK-MB, ng/mL	−0.05 (−0.08–0.00)	1.05 (1.01–1.07)	0.990 (0.980–0.995)	0.01 (−0.01–0.05)	1.00 (0.98–1.02)	0.975 (0.952–0.987)
fT4, ng/dL	0.07 (−0.01–0.15) *	0.91 (0.83–0.99) *	0.958 (0.921–0.978) *	−0.01(−0.13–0.07)	1.00 (0.91–1.12)	0.919 (0.849–0.958)
TSH, μIU/mL	0.01 (−0.04–0.05) *	0.99 (0.96–1.02) *	0.999 (0.998–0.999) *	0.02 (−0.01–0.06) *	0.98 (0.95–1.00) *	0.999 (0.998–0.999) *
HBsAg, S/CO	−0.03 (−0.13–0.08)	1.02 (0.67–1.33)	0.394 (0.084–0.634)	0.02 (−0.13–0.08)	1.00 (0.82–1.50)	0.526 (0.248–0.724)
Anti-HCV, S/CO	0.00 (−0.01–0.00)	1.00 (1.00–1.14)	0.973 (0.949–0.986)	0.00 (−0.01–0.00)	1.00 (1.00–1.14)	0.948 (0.902–0.973)
HIV Ag/Ab, S/CO	−0.01 (−0.05–0.01)	1.00 (0.76–1.50)	0.601 (0.348–0.773)	0.00 (−0.03–0.01)	1.00 (0.78–1.38)	0.705 (0.497–0.836)

* Refer to Table 1 for the same annotation. Abbreviations: see Table 1.

**Table 3:** Stability test comparing VQ-Tube SST with 5 min and 30 min clotting times.

Measurand	VQ5	VQ30	*p*	Bias (%) (95% CI)	Intercept (95% CI)	Slope (95% CI)	ρ (95% CI)
	Mean/Median						
AST, U/L	21.5 (18.0–25.6)	21.0 (18.0–25.0)	0.09	0.00 (0.00–3.45)	0.00 (0.00–0.00)	1.00 (1.00–1.00)	0.989 (0.980–0.995)
ALT, U/L	14.0 (11.4–21.0)	14.0 (12.0–22.0)	0.72	−0.04 (−2.18–2.09) *	0.00 (−0.72–0.50)	1.00 (1.00–1.00)	0.974 (0.949–0.986)
GGT, U/L	13.5 (12.0–30.1)	13.0 (12.0–30.7)	0.02	0.00 (0.00–0.00)	0.00 (0.00–0.00)	1.00 (1.00–1.00)	0.996 (0.992–0.998)
ALP, U/L	55.0 (47.0–82.0)	55.0 (46.8–82.0)	0.31	0.00 (0.00–0.00)	0.00 (0.00–0.00)	1.00 (1.00–1.00)	0.999 (0.997–0.999)
TP, g/dL	7.2 (6.9–7.4)	7.2 (6.9–7.4)	0.93	0.00 (0.00–0.00)	0.00 (−0.90–0.00)	1.00 (1.00–1.13)	0.985 (0.971–0.992)
Alb, g/dL	4.5 (4.4–4.7)	4.5 (4.4–4.7)	0.08	0.00 (0.00–0.00)	0.00 (0.00–0.00)	1.00 (1.00–1.00)	0.891 (0.798–0.942)
TB, mg/dL	0.62 (0.47–0.69)	0.61 (0.47–0.70)	0.47	0.00 (−1.19–0.00)	0.00 (−0.01–0.02)	1.00 (0.97–1.00)	0.992 (0.985–0.996)
DB, mg/dL	0.12 (0.09–0.14)	0.12 (0.09–0.13)	0.25	0.00 (−5.56–0.00)	0.00 (−0.01–0.01)	1.00 (0.88–1.00)	0.968 (0.939–0.983)
BUN, mg/dL	12.8 (10.4–16.6)	12.9 (10.3–16.6)	0.66	0.00 (0.00–0.64)	0.00 (−0.11–0.16)	1.00 (0.99–1.01)	0.999 (0.997–0.999)
Cr, mg/dL	0.75 (0.66–0.82)	0.74 (0.66–0.82)	0.10	−0.40 (−1.03–0.23) *	0.00 (−0.01–0.04)	1.00 (0.94–1.00)	0.990 (0.980–0.995)
Cholesterol, mg/dL	187.9 ± 35.3 *	188.3 ± 35.2 *	0.24 *	0.22 (−0.13–0.58) *	0.92 (−2.49–4.33) *	1.00 (0.98–1.02) *	0.998 (0.997–0.999) *
LDL, mg/dL	106.6 ± 31.3 *	106.7 ± 31.3 *	0.63	0.00 (−0.68–0.80)	0.02 (−1.61–1.65) *	1.00 (0.99–1.02) *	0.999 (0.998–1.000) *
HDL, mg/dL	63.4 ± 12.8 *	63.7 ± 12.8 *	0.35	0.00 (0.00–1.19)	0.47 (−1.29–2.22) *	1.00 (0.97–1.03) *	0.993 (0.986–0.996) *
TG, mg/dL	102.0 (62.8–158.8)	102.0 (62.4–159.2)	0.06	0.00 (−0.87–0.00)	0.00 (−1.00–0.51)	1.00 (0.99–1.01)	1.000 (1.000–1.000)
Glucose, mg/dL	100.0 (97.0–115.0)	99.5 (93.0–110.0)	<0.0001 *	−3.08 (−3.76–−2.40) *	−3.00 (−5.00–0.86)	1.00 (0.97–1.02)	0.982 (0.965–0.991)
UA, mg/dL	4.60 (3.99–5.29)	4.59 (3.97–5.29)	<0.0001 *	−0.68 (−0.96–−0.39) *	−0.03 (−0.07–0.03)	1.00 (0.99–1.01)	0.996 (0.993–0.998)
LDH, U/L	147.0 (129.4–168.0)	148.5 (137.0–177.2)	<0.0001 *	4.60 (2.71–6.49) *	10.91 (−1.79–29.0)	0.97 (0.84–1.07)	0.934 (0.876–0.966)
CK, U/L	99.5 (76.0–151.7)	97.0 (76.0–150.3)	0.24 *	−0.64 (−1.28–−0.002) *	−1.00 (−3.12–1.02)	1.00 (0.98–1.03)	0.997 (0.995–0.999)
Amylase, U/L	61.0 (44.8–71.2)	60.5 (44.8–71.2)	0.76 *	0.00 (0.00–1.52)	0.00 (−1.18–1.11)	1.00 (0.98–1.03)	0.994 (0.988–0.997)
Lipase, U/L	25.5 (15.4–33.0)	25.5 (15.4–32.0)	0.34	0.00 (0.00–0.00)	0.00 (−0.27–0.00)	1.00 (1.00–1.02)	0.995 (0.990–0.997)
TIBC, μg/dL	337.9 ± 51.5 *	338.1 ± 50.7 *	0.53	0.29 (0.00–0.38)	5.11 (−2.33–12.54) *	0.99 (0.96–1.01) *	0.997 (0.995–0.999) *
Na, mmol/L	138.9 ± 2.2 *	140.0 (138.0–140.0)	0.051	0.00 (0.00–0.71)	0.00 (−27.60–1.00)	1.00 (1.00–1.20)	0.850 (0.728–0.920)
K, mmol/L	4.0 (3.9–4.3)	4.15 ± 0.32 *	0.01 *	0.00 (0.00–2.50)	0.00 (−0.70–0.10)	1.00 (1.00–1.20)	0.945 (0.897–0.971)
Cl, mmol/L	104.0 (103.0–105.0)	104.0 (103.0–105.0)	0.58	0.00 (0.00–0.00)	0.00 (−10.40–0.00)	1.00 (1.00–1.10)	0.884 (0.787–0.939)
Mg, mg/dL	0.9 (0.8–0.9)	0.9 (0.8–0.9)	0.31	0.00 (0.00–0.00)	0.00 (0.00–0.00)	1.00 (1.00–1.00)	0.759 (0.579–0.868)
Ca, mg/dL	9.70 (9.44–9.90)	9.70 (9.50–10.00)	0.01	0.00 (0.00–1.00)	0.00 (0.00–0.10)	1.00 (1.00–1.00)	0.978 (0.957–0.989)
P, mg/dL	3.87 ± 0.44 *	3.88 ± 0.46 *	0.46 *	0.27 (−0.30–0.77)	−0.15 (−0.44–0.14) *	1.04 (0.97–1.11) *	0.993 (0.987–0.996) *
Fe, µg/dL	90.9 ± 36.3 *	91.9 ± 36.0 *	0.001	0.74 (0.00–1.22)	1.77 (0.43–3.12) *	0.99 (0.98–1.00) *	0.998 (0.996–0.999) *
CRP, mg/L	0.60 (0.26–1.01)	0.57 (0.26–1.02)	0.49	0.00 (−1.54–0.00)	0.00 (−0.01–0.002) *	1.00 (0.99–1.02)	0.996 (0.992–0.998)

* Refer to Table 1 for the same annotation. Abbreviations: VQ5, VQ-Tube SST with 5 min clotting time; VQ30, VQ-Tube SST with 30 min clotting time; others in Table 1.

**Table 4:** Comparison between V-Tube K_2_EDTA and BD K_2_EDTA.

Measurand	BD K_2_EDTA	V-Tube K_2_EDTA	*p*	Bias (%) (95% CI)	Ricos (%)	EFLM (%)	Intercept (95% CI)	Slope (95% CI)	ρ (95% CI)
RBC, 10^12^/L	4.4 ± 0.5 *	4.4 ± 0.5 *	0.95 *	0.01 (−0.33–0.35) *	1.7	1.9	−0.04 (−0.17–0.08) *	1.01 (0.98–1.04) *	0.995 (0.991–0.997) *
Hb, g/dL	12.9 (12.5–13.9)	13.0 (12.5–13.8)	0.87	0.00 (0.00–0.00)	1.84	1.7	0.00 (0.00–0.00)	1.00 (1.00–1.00)	0.994 (0.989–0.997)
Hct, %	40.0 ± 3.6 *	40.7 ± 3.6 *	<0.0001 *	1.74 (1.39–2.09) *	1.74	1.5	0.56 (−1.04–2.16) *	1.00 (0.96–1.04) *	0.993 (0.987–0.996) *
MCV, fL	92.1 ± 3.6 *	93.7 ± 3.8 *	<0.0001	1.87 (1.55–2.13)	1.26	1.0	−5.12 (−13.82–3.59) *	1.07 (0.98–1.17) *	0.982 (0.966–0.991) *
MCH, pg	30.5 ± 1.2 *	30.5 ± 1.2 *	0.74 *	0.06 (−0.29–0.41) *	1.35	1.2	0.01 (−2.58–2.59) *	1.00 (0.92–1.09) *	0.963 (0.930–0.980) *
MCHC, g/dL	33.2 ± 0.7 *	32.6 ± 0.8 *	<0.0001 *	−1.63 (−2.01–−1.26) *	0.4	0.4	−3.97 (−12.25–4.32) *	1.10 (0.85–1.35) *	0.868 (0.762–0.928) *
RDW, %	12.9 ± 0.7 *	12.9 (12.3–13.2)	0.03	0.00 (0.00–0.73)	1.7	1.2	−0.61 (−1.60–0.05)	1.05 (1.00–1.13)	0.968 (0.939–0.983)
Reticulocyte, 10^12^/L	0.07 (0.05–0.08)	0.06 (0.05–0.08)	0.51	1.06 (−1.29–3.42) *	7.8	7.2	0.00 (0.00–0.01)	1.01 (0.92–1.06)	0.965 (0.934–0.982)
IRF, %	6.3 ± 2.9 *	6.1 (4.6–8.3)	0.06 *	6.83 (1.30–12.16)	N/A	N/A	0.30 (−0.63–1.11)	1.00 (0.88–1.17)	0.874 (0.768–0.933)
WBC, 10^9^/L	5.74 (5.00–7.20)	5.75 (5.07–7.34)	0.29 *	0.73 (−0.11–1.56) *	6.05	5.1	0.18 (0.04–0.37)	0.97 (0.94–1.00)	0.987 (0.974–0.993)
Neutrophil, 10^9^/L	3.00 (2.62–4.05)	3.03 (2.65–4.10)	0.25 *	0.75 (−0.16–1.66) *	9.25	7.2	0.07 (−0.03–0.18)	0.99 (0.95–1.01)	0.991 (0.982–0.995)
Lymphocyte, 10^9^/L	2.04 (1.78–2.48)	2.06 (1.75–2.52)	0.60 *	0.63 (−0.67–1.93) *	9.19	6.3	0.09 (−0.04–0.20)	0.96 (0.91–1.03)	0.989 (0.979–0.994)
Monocyte, 10^9^/L	0.40 (0.33–0.50)	0.42 (0.33–0.52)	0.75	0.37 (−2.61–3.34) *	13.2	6.7	−0.01 (−0.06–0.03)	1.03 (0.94–1.18)	0.938 (0.884–0.968)
Eosinophil, 10^9^/L	0.09 (0.06–0.14)	0.09 (0.06–0.15)	0.10 *	6.18 (0.09–12.27) *	19.8	17.3	0.01 (0.00–0.01)	1.01 (0.96–1.06)	0.948 (0.902–0.973)
Basophil, 10^9^/L	0.03 (0.03–0.06)	0.04 (0.02–0.06)	0.18	3.05 (−1.12–10.91)	15.4	7.8	0.00 (0.00–0.00)	1.10 (0.96–1.22)	0.923 (0.855–0.959)
Platelet, 10^9^/L	278.7 ± 54.2 *	276.6 ± 53.2 *	0.054 *	−0.67 (−1.50–0.16) *	5.9	5.0	2.98 (−6.46–12.42) *	0.98 (0.95–1.01) *	0.992 (0.986–0.996) *
Plateletcrit, %	0.28 ± 0.05 *	0.28 ± 0.05 *	0.13	0.00 (−3.45–0.00)	N/A	3.7	0.00 (−0.02–0.02) *	1.00 (0.93–1.07) *	0.969 (0.941–0.983) *
MPV, fL	10.2 ± 0.7 *	10.2 ± 0.7 *	0.31	0.00 (−0.92–1.01)	2.29	1.9	0.07 (−0.80–0.94) *	1.00 (0.91–1.08) *	0.968 (0.941–0.983) *
PDW, fL	12.3 (11.4–13.0)	12.2 ± 1.3 *	0.76	0.00 (−2.29–2.54)	N/A	3.2	0.00 (−2.10–2.53)	1.00 (0.79–1.18)	0.834 (0.702–0.911)
ESR, mm/h	7.0 (3.0–15.6)	5.0 (2.4–11.2)	0.002	−5.71 (−25.00–0.00)	5 ^†^	5 ^†^	0.40 (−0.14–1.60)	0.80 (0.60–0.95)	0.835 (0.703–0.912)
HbA1c, %	5.4 (5.1–5.7)	5.5 (5.2–5.8)	0.053	1.74 (0.00–1.85)	1.5	1.4	0.10 (0.00–0.63)	1.00 (0.89–1.00)	0.946 (0.898–0.972)

* Refer to Table 1 for the same annotations. ^†^ Arbitrarily defined as half of the total allowable error recommended by the manufacturer. Abbreviations: RBC, erythrocyte count; Hb, hemoglobin; Hct, hematocrit; MCV, mean corpuscular volume; MCH, mean corpuscular hemoglobin; MCHC, mean corpuscular hemoglobin concentration; RDW, red cell distribution width; IRF, immature reticulocyte fraction; WBC, leukocyte count; PLT, thrombocyte count; MPV, mean platelet volume; PDW, platelet distribution width; ESR, erythrocyte sedimentation rate; HbA1c, hemoglobin A1c.

## Data Availability

All the data used to support the findings of this study are included within the article, and the research data will be made available upon request to the corresponding author.
