# Correction: Kang et al. Comparative Evaluation of Serum Separator V-Tube™, VQ-Tube™, and K_2_EDTA V-Tube™ with Becton Dickinson Tubes for Chemistry, Immunology, and Hematology Examinations. *Diagnostics* 2025, *15*, 1775

**DOI:** 10.3390/diagnostics16152446

**Published:** 2026-08-03

**Authors:** Takho Kang, Seung Gyu Yun, Myung-Hyun Nam, Yunjung Cho, Minjeong Nam

**Affiliations:** Department of Laboratory Medicine, Korea University Anam Hospital, Korea University College of Medicine, Seoul 02841, Republic of Korea

## Author Information

In the original publication [1], the ORCID link of Yunjung Cho is missing. The ORCID link is https://orcid.org/0000-0002-6942-8385.

The affiliation information is incomplete. The correction information is Department of Laboratory Medicine, Korea University Anam Hospital, Korea University College of Medicine, Seoul 02841, Republic of Korea.

## Errors in Figure/Table

In the original publication [1], there were mistakes in Figures 1–3 and Tables 1–3, including figure legends and table captions, and the units in tables.

Figure 1A represented the glucose bias (which was calculated as the median), its 95% CI, and allowable difference (which was quoted from EFLM desirable bias), plotted with the Analyze-it software. Figure 1B represented the LDH bias (which was calculated as the mean), its 95% CI, and allowable difference (EFLM desirable bias). However, original Figure 1A was labeled as mean, and Figure 1B contained a formatting error where the image overlapped with another graphic. A correction needs to be made for Figure 1A,B. The original caption for Figure 1 contains a statistical inaccuracy. While a positive bias generally indicates higher values, it reflects the systematic tendency across all measurements rather than the higher values themselves.

To ensure consistency with the corrected figures and remove the unnecessary abbreviations note, a correction also needs to be made for Figure 2.

**Figure 2 diagnostics-16-02446-f002:**
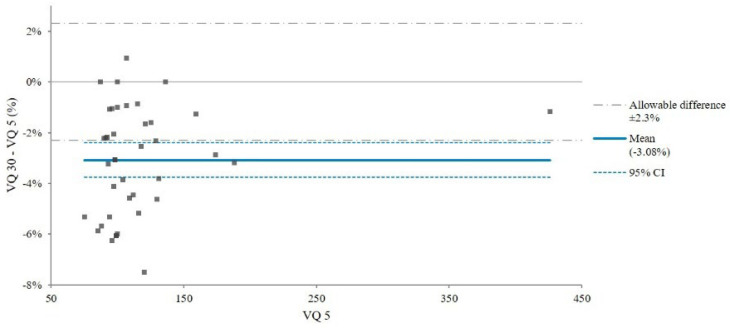
Difference plot for stability assessment of glucose between VQ-Tube SST with 5 min and 30 min clotting time. Bias and 95% CI (−3.08%, −3.76–−2.40%) did not overlap with the EFLM desirable bias of 2.3%.

Figure 3A was plotted to represent the MCV’s bias (which was calculated as the median), its 95% CI, and allowable difference (EFLM desirable bias). However, the original Figure 3A was mistakenly labeled as mean. A correction needs to be made for Figure 3A. To ensure consistency with the corrected figure, a correction also needs to be made for Figure 3B.

The units in Table 1 for certain measurands were incorrectly stated, which needs to be corrected.

**Table 1 diagnostics-16-02446-t001:** Comparative analysis of chemistry and immunology measurands between BD SST, V-Tube SST, and VQ-tube SST.

Measurand	BD SST (Mean/Median)	V-Tube SST			VQ-Tube SST			Ricos (%)	EFLM (%)
		Mean/Median	*p*	Bias (%) (95% CI)	Mean/Median	*p*	Bias (%) (95% CI)		
AST, U/L	21.5 (18.0–25.6)	21.5 (18.0–25.0)	0.34	0.00 (−2.86–0.00)	21.5 (18.0–25.6)	0.34	0.00 (0.00–0.00)	6.54	5.3
ALT, U/L	14.0 (12.4–21.6)	15.0 (12.0–21.0)	0.79	0.00 (0.00–0.00)	14.0 (11.4–21.0)	0.37	0.00 (0.00–0.00)	11.48	8.1
GGT, U/L	13.0 (11.0–30.5)	13.0 (11.0–30.1)	0.21	0.00 (0.00–0.00)	13.5 (12.0–30.1)	1.00	0.00 (0.00–0.00)	11.06	11.5
ALP, U/L	54.5 (47.0–81.0)	55.0 (46.4–80.0)	0.25	−0.26 (−0.82–0.29) *	55.0 (47.0–82.0)	0.64	0.00 (0.00–0.00)	6.72	5.5
TP, g/dL	7.1 (6.8–7.4)	7.1 (6.9–7.4)	0.11	0.00 (0.00–1.30)	7.2 (6.9–7.4)	0.04	0.00 (0.00–1.43)	1.36	1.3
Alb, g/dL	4.5 (4.4–4.7)	4.5 (4.4–4.6)	0.85	0.00 (0.00–0.00)	4.5 (4.4–4.7)	0.66	0.00 (0.00–0.00)	1.43	1.2
TB, mg/dL	0.61 ± 0.18 *	0.61 (0.48–0.68)	0.23	0.00 (0.00–1.70)	0.62 (0.47–0.69)	0.03	1.56 (0.00–1.85)	8.95	8.0
DB, mg/dL	0.12 ± 0.04 *	0.12 ± 0.04 *	0.90 *	0.00 (0.00–5.56)	0.12 ± 0.04 *	0.69	0.00 (0.00–0.00)	14.2	N/A
BUN, mg/dL	12.8 (10.3–16.6)	12.9 (10.4–16.6)	0.21 *	0.24 (−0.16–0.64) *	12.8 (10.4–16.6)	0.48	0.00 (0.00–0.65)	5.57	6.0
Cr, mg/dL	0.75 (0.66–0.83)	0.75 (0.66–0.83)	0.02 *	−1.01 (−1.79–−0.22) *	0.75 (0.66–0.82)	0.39	−0.18 (−0.86–0.50) *	3.96	4.2
Cholesterol, mg/dL	187.3 ± 34.6 *	187.5 ± 35.7 *	0.92	0.02 (−0.38–0.43) *	187.9 ± 35.3 *	0.34	0.28 (−0.16–0.73) *	4.1	4.1
LDL, mg/dL	105.8 ± 31.2 *	106.1 ± 31.2 *	0.14	0.00 (0.00–1.01)	106.6 ± 31.3 *	0.009	0.00 (0.00–0.99)	5.46	5.6
HDL, mg/dL	61.9 ± 13.0 *	63.3 ± 12.6 *	<0.0001 *	2.49 (1.52–3.46) *	63.4 ± 12.8 *	<0.0001 *	2.62 (1.64–3.61) *	5.61	5.5
TG, mg/dL	101.0 (61.8–157.6)	102.0 (63.0–157.2)	0.22	0.00 (0.00–0.88)	102.0 (62.8–158.8)	<0.0001 *	1.07 (0.54–1.60) *	9.57	9.9
Glucose, mg/dL	98.0 (90.8–115.0)	99.5 (90.4–114.9)	0.14	0.00 (0.00–1.05)	100.0 (94.8–120.6)	<0.0001 *	3.45 (2.11–6.67)	1.8	2.3
UA, mg/dL	4.63 (4.03–5.32)	4.61 (4.01–5.29)	0.0003 *	−0.56 (−0.85–−0.27) *	4.60 (3.99–5.29)	0.03 *	−0.33 (−0.61–−0.05) *	4.87	N/A
LDH, U/L	157.5 (145.0–182.1)	148.0 (134.4–171.6)	0.0002	−4.68 (−6.96–−2.41) *	147.0 (129.4–168.0)	<0.0001 *	−7.87 (−10.25–−5.49) *	4.3	3.1
CK, U/L	101.0 (76.0–151.3)	98.0 (75.0–150.3)	0.67 *	−0.14 (−1.00–0.72) *	99.5 (76.0–151.7)	0.70	0.00 (−0.54–1.02)	11.5	8.9
Amylase, U/L	60.0 (44.8–71.0)	61.0 (45.3–70.6)	0.03	0.74 (0.04–1.43) *	61.0 (44.8–71.2)	0.24	0.41 (−0.35–1.17) *	7.4	7.5
Lipase, U/L	26.0 (15.0–31.6)	25.5 (15.0–32.0)	1.00	0.00 (0.00–0.00)	25.5 (15.4–33.0)	0.41	0.00 (0.00–0.00)	11.31	6.3
TIBC, μg/dL	337.1 ± 50.7 *	337.5 ± 51.3 *	0.78	0.00 (−0.34–0.32)	337.9 ± 51.5 *	0.22 *	0.24 (−0.17–0.65) *	N/A	N/A
Na, mmol/L	139.3 ± 2.1 *	140.0 (138.0–141.0)	0.61	0.00 (0.00–0.00)	138.9 ± 2.2 *	0.009	−0.35 (−0.71–0.00)	0.23	0.2
K, mmol/L	4.1 (3.9–4.4)	4.2 (3.9–4.4)	0.003	2.44 (0.00–2.50)	4.0 (3.9–4.3)	0.07	0.00 (−2.27–0.00)	1.81	1.7
Cl, mmol/L	104.0 (103.0–105.0)	104.0 (103.0–105.0)	0.60	0.00 (0.00–0.00)	104.0 (103.0–105.0)	0.87	0.00 (0.00–0.00)	0.5	0.4
Mg, mg/dL	0.8 (0.8–0.9)	0.9 (0.8–0.9)	0.13	0.00 (0.00–0.00)	0.9 (0.8–0.9)	0.008	0.00 (0.00–0.00)	1.8	1.6
Ca, mg/dL	9.7 (9.4–9.9)	9.7 (9.5–9.9)	0.02	0.50 (0.00–1.04)	9.7 (9.4–9.9)	0.43	0.00 (0.00–1.02)	0.82	0.7
P, mg/dL	3.85 ± 0.46 *	3.86 ± 0.46 *	0.04 *	0.41 (0.03–0.80) *	3.87 ± 0.44 *	0.02 *	0.56 (−0.24–1.12)	3.38	3.3
Fe, µg/dL	91.1 ± 36.1 *	90.8 ± 36.0 *	0.21	0.00 (−0.74–0.00)	90.9 ± 36.3 *	0.47 *	0.00 (−0.81–0.00)	8.8	9.6
CRP, mg/L	0.60 (0.26–1.03)	0.58 (0.25–1.01)	0.37	0.00 (−1.49–0.00)	0.60 (0.26–1.01)	0.23	0.00 (0.00–2.61)	21.8	23.4
PCT, ng/mL	0.03 (0.02–0.04)	0.03 (0.02–0.04)	0.80	0.00 (0.00–0.00)	0.03 (0.02–0.03)	0.30	0.00 (0.00–0.00)	N/A	N/A
AFP, ng/mL	2.66 (1.86–3.52)	2.70 (1.83–3.54)	0.02	1.31 (0.17–2.45) *	2.71 (1.87–3.50)	0.004 *	2.03 (0.84–2.86)	11.8	13.8
CEA, ng/mL	1.42 (1.00–2.07)	1.41 (0.98–2.06)	0.19	−0.45 (−1.24–0.34) *	1.45 (0.97–2.09)	0.80 *	0.00 (−1.12–1.49)	14.3	14.9
PSA, ng/mL	0.01 (0.01–0.18)	0.01 (0.01–0.20)	0.63	0.00 (0.00–0.00)	0.01 (0.01–0.20)	0.84	0.00 (0.00–0.00)	18.7	10.6
Troponin-T, ng/mL	0.003 (0.003–0.005)	0.003 (0.003–0.005)	0.25	0.00 (0.00–0.00)	0.003 (0.003–0.004)	0.31	0.00 (0.00–0.00)	23.7	8.2
CK-MB, ng/mL	1.32 (1.01–1.86)	1.31 (1.03–1.87)	0.01	0.82 (−0.45–2.27)	1.33 (1.04–1.86)	0.02 *	0.93 (0.00–2.51)	14.88	N/A
fT4, ng/dL	0.94 ± 0.11 *	0.93 ± 0.10 *	0.002 *	−1.58 (−2.67–−0.49) *	0.91 (0.83–1.02)	0.002 *	−1.94 (−3.14–−0.74) *	3.3	2.3
TSH, μIU/mL	1.78 ± 0.91 *	1.76 ± 0.91 *	0.04	−0.63 (−1.55–0.00)	1.76 ± 0.89 *	0.03	0.00 (−1.14–0.40)	7.8	10.0
HBsAg, S/CO	0.33 (0.29–0.35)	0.28 (0.26–0.32)	0.001	−8.86 (−12.93–−4.80) *	0.30 (0.27–0.32)	0.005	−6.74 (−10.55–−2.93) *	N/A	N/A
Anti-HCV, S/CO	0.06 (0.05–0.07)	0.06 (0.04–0.07)	0.27	0.00 (0.00–0.00)	0.05 (0.04–0.07)	0.08	0.00 (0.00–0.00)	N/A	N/A
HIV Ag/Ab, S/CO	0.07 (0.07–0.09)	0.07 (0.06–0.09)	0.008	−6.80 (−12.92–−0.68) *	0.07 (0.06–0.08)	0.08	0.00 (−11.11–0.00)	N/A	N/A

* Parametric analyses (mean ± SD, paired *t*-test, Deming regression, and Pearson correlation) were applied; otherwise, nonparametric analyses (median [1st and 3rd quartile], Wilcoxon signed rank test, Passing–Bablok regression, and Spearman’s correlation) were applied. Abbreviations: SST, serum separator tube; CI, confidence interval; AST, aspartate aminotransferase; ALT, alanine aminotransferase; GGT, gamma-glutamyl transferase; ALP, alkaline phosphatase; TP, total protein; Alb, albumin; TB, total bilirubin; DB, direct bilirubin; BUN, blood urea nitrogen; Cr, creatinine; Cholesterol, total cholesterol; LDL, low-density lipoprotein cholesterol; HDL, high-density lipoprotein cholesterol; TG, triglycerides; UA, uric acid; LDH, lactate dehydrogenase; CK, creatine kinase; TIBC, total iron-binding capacity; Na, sodium; K, potassium; Cl, chloride; Mg, magnesium; Ca, calcium; P, phosphorus; Fe, iron; CRP, C-reactive protein; PCT, procalcitonin; AFP, alpha-fetoprotein; CEA, carcinoembryonic antigen; PSA, prostate-specific antigen; CK-MB, creatine kinase-MB; fT4, free thyroxine; TSH, thyroid-stimulating hormone; HBsAg, hepatitis B surface antigen; Anti-HCV, hepatitis C virus antibody; HIV Ag/Ab, human immunodeficiency virus antigen/antibody.

The units in Table 2 for certain measurands were incorrectly stated, which needs to be corrected.

**Table 2 diagnostics-16-02446-t002:** Correlation analysis between BD SST, V-Tube SST, and VQ-tube SST for chemistry and immunology measurands.

Measurand	BD and V-Tube SST			BD and VQ-Tube SST		
	Intercept (95% CI)	Slope (95% CI)	ρ (95% CI)	Intercept (95% CI)	Slope (95% CI)	ρ (95% CI)
AST, U/L	0.00 (−0.50–1.83)	1.00 (0.90–1.00)	0.982 (0.966–0.991)	0.00 (0.00–1.50)	1.00 (0.92–1.00)	0.986 (0.974–0.993)
ALT, U/L	0.00 (0.00–0.76)	1.00 (0.96–1.00)	0.983 (0.968–0.991)	0.00 (0.00–0.00)	1.00 (1.00–1.00)	0.995 (0.990–0.997)
GGT, U/L	0.00 (0.00–0.00)	1.00 (1.00–1.00)	0.986 (0.973–0.993)	0.00 (0.00–0.00)	1.00 (1.00–1.00)	0.986 (0.974–0.993)
ALP, U/L	0.00 (−1.00–0.83)	1.00 (0.98–1.00)	0.997 (0.995–0.999)	0.00 (−1.71–0.00)	1.00 (1.00–1.02)	0.997 (0.994–0.998)
TP, g/dL	0.00 (0.00–1.00)	1.00 (1.00–1.00)	0.976 (0.954–0.988)	0.00 (0.00–1.06)	1.00 (0.86–1.00)	0.967 (0.936–0.983)
Alb, g/dL	0.00 (0.00–0.00)	1.00 (1.00–1.00)	0.908 (0.828–0.951)	0.00 (0.00–0.00)	1.00 (1.00–1.00)	0.912 (0.836–0.954)
TB, mg/dL	0.00 (−0.01–0.02)	1.00 (0.97–1.03)	0.988 (0.977–0.994)	0.01 (−0.02–0.02)	1.00 (0.98–1.05)	0.988 (0.977–0.994)
DB, mg/dL	0.00 (−0.02–0.02) *	1.00 (0.86–1.14) *	0.936 (0.881–0.966) *	0.00 (−0.02–0.01) *	1.04 (0.89–1.18) *	0.953 (0.913–0.975) *
BUN, mg/dL	0.05 (−0.12–0.10)	1.00 (1.00–1.01)	0.997 (0.995–0.999)	0.00 (−0.21–0.22)	1.00 (0.98–1.02)	0.997 (0.994–0.998)
Cr, mg/dL	−0.02 (−0.05–0.00)	1.02 (1.00–1.06)	0.991 (0.982–0.995)	0.00 (−0.02–0.01)	1.00 (1.00–1.03)	0.988 (0.978–0.994)
Cholesterol, mg/dL	−5.86 (−10.62–−1.09) *	1.03 (1.01–1.06) *	0.998 (0.996–0.999) *	−3.53 (−8.24–1.17) *	1.02 (1.00–1.05) *	0.997 (0.995–0.999) *
LDL, mg/dL	0.41 (−1.53–2.34) *	1.00 (0.98–1.02) *	0.999 (0.998–0.999) *	0.44 (−1.54–2.43) *	1.00 (0.98–1.02) *	0.998 (0.997–0.999) *
HDL, mg/dL	2.95 (0.41–5.50) *	0.97 (0.94–1.01) *	0.992 (0.984–0.996) *	2.07 (−0.77–4.90) *	0.99 (0.94–1.04) *	0.990 (0.982–0.995) *
TG, mg/dL	0.00 (−0.54–1.00)	1.00 (1.00–1.01)	0.999 (0.999–1.000)	0.61 (−0.46–1.11)	1.01 (1.00–1.02)	0.999 (0.999–1.000)
Glucose, mg/dL	0.00 (−4.12–1.00)	1.00 (1.00–1.04)	0.993 (0.986–0.996)	4.50 (−2.40–9.17)	1.00 (0.96–1.07)	0.960 (0.923–0.979)
UA, mg/dL	−0.02 (−0.06–0.03)	1.00 (0.99–1.01)	0.993 (0.986–0.996)	−0.01 (−0.06–0.04)	1.00 (0.99–1.01)	0.995 (0.990–0.997)
LDH, U/L	−23.71 (−42.42–−8.00)	1.10 (1.00–1.21)	0.894 (0.803–0.944)	−20.58 (−45.50–−0.50)	1.07 (0.91–1.21)	0.858 (0.741–0.924)
CK, U/L	0.00 (−2.69–2.66)	1.00 (0.97–1.02)	0.996 (0.992–0.998)	0.00 (−2.31–2.08)	1.00 (0.99–1.02)	0.993 (0.987–0.996)
Amylase, U/L	0.00 (0.00–1.00)	1.00 (1.00–1.00)	0.997 (0.994–0.998)	0.00 (−1.02–1.00)	1.00 (1.00–1.02)	0.997 (0.994–0.998)
Lipase, U/L	0.00 (−1.08–0.00)	1.00 (1.00–1.04)	0.995 (0.990–0.997)	0.00 (0.00–0.71)	1.00 (0.97–1.00)	0.993 (0.986–0.996)
TIBC, μg/dL	−3.10 (−10.89–4.70) *	1.01 (0.99–1.03) *	0.998 (0.996–0.999) *	−4.02 (−12.68–4.65) *	1.01 (0.99–1.04) *	0.997 (0.994–0.998) *
Na, mmol/L	0.00 (−46.67–0.00)	1.00 (1.00–1.33)	0.954 (0.913–0.976)	−6.29 (−25.09–12.52) *	1.04 (0.91–1.18) *	0.943 (0.893–0.969) *
K, mmol/L	0.10 (−0.72–0.60)	1.00 (0.88–1.20)	0.933 (0.874–0.965)	0.00 (−0.05–1.01)	1.00 (0.75–1.00)	0.852 (0.732–0.921)
Cl, mmol/L	0.00 (0.00–0.00)	1.00 (1.00–1.00)	0.887 (0.792–0.940)	0.00 (0.00–0.00)	1.00 (1.00–1.00)	0.930 (0.868–0.963)
Mg, mg/dL	0.00 (0.00–0.00)	1.00 (1.00–1.00)	0.864 (0.753–0.928)	0.00 (0.00–0.00)	1.00 (1.00–1.00)	0.788 (0.626–0.885)
Ca, mg/dL	0.05 (0.00–0.10)	1.00 (1.00–1.00)	0.941 (0.889–0.969)	0.00 (−1.44–0.10)	1.00 (1.00–1.14)	0.927 (0.863–0.962)
P, mg/dL	0.00 (−0.10–0.10) *	1.00 (0.98–1.03) *	0.995 (0.990–0.997) *	0.16 (−0.09–0.41) *	0.96 (0.90–1.03) *	0.992 (0.984–0.996) *
Fe, µg/dL	0.15 (−0.62–0.93) *	1.00 (0.99–1.00) *	1.00 (0.999–1.000) *	−0.72 (−1.72–0.28) *	1.01 (0.99–1.02) *	0.999 (0.998–1.000) *
CRP, mg/L	0.00 (−0.01–0.01)	0.99 (0.98–1.00)	0.997 (0.994–0.998)	0.00 (0.00–0.01)	1.00 (0.99–1.02)	0.996 (0.992–0.998)
PCT, ng/mL	0.00 (0.00–0.00)	1.00 (1.00–1.00)	0.667 (0.441–0.814)	0.00 (0.00–0.00)	1.00 (1.00–1.00)	0.748 (0.562–0.862)
AFP, ng/mL	0.00 (−0.06–0.06)	1.01 (0.99–1.04)	0.993 (0.987–0.997)	0.02 (−0.03–0.08)	1.01 (0.98–1.03)	0.992 (0.985–0.996)
CEA, ng/mL	−0.01 (−0.04–0.02)	1.00 (0.98–1.02)	0.998 (0.996–0.999)	−0.01 (−0.05–0.01)	1.01 (0.99–1.03)	0.998 (0.995–0.999)
PSA, ng/mL	0.00 (0.00–0.00)	1.00 (1.00–1.03)	1.000 (1.000–1.000)	0.00 (0.00–0.00)	1.00 (1.00–1.00)	1.000 (1.000–1.000)
Troponin-T, ng/mL	0.00 (0.00–0.00)	1.00 (1.00–1.03)	1.000 (0.999–1.000)	0.00 (0.00–0.00)	1.00 (1.00–1.00)	0.883 (0.785–0.938)
CK-MB, ng/mL	−0.05 (−0.08–0.00)	1.05 (1.01–1.07)	0.990 (0.980–0.995)	0.01 (−0.01–0.05)	1.00 (0.98–1.02)	0.975 (0.952–0.987)
fT4, ng/dL	0.07 (−0.01–0.15) *	0.91 (0.83–0.99) *	0.958 (0.921–0.978) *	−0.01(−0.13–0.07)	1.00 (0.91–1.12)	0.919 (0.849–0.958)
TSH, μIU/mL	0.01 (−0.04–0.05) *	0.99 (0.96–1.02) *	0.999 (0.998–0.999) *	0.02 (−0.01–0.06) *	0.98 (0.95–1.00) *	0.999 (0.998–0.999) *
HBsAg, S/CO	−0.03 (−0.13–0.08)	1.02 (0.67–1.33)	0.394 (0.084–0.634)	0.02 (−0.13–0.08)	1.00 (0.82–1.50)	0.526 (0.248–0.724)
Anti-HCV, S/CO	0.00 (−0.01–0.00)	1.00 (1.00–1.14)	0.973 (0.949–0.986)	0.00 (−0.01–0.00)	1.00 (1.00–1.14)	0.948 (0.902–0.973)
HIV Ag/Ab, S/CO	−0.01 (−0.05–0.01)	1.00 (0.76–1.50)	0.601 (0.348–0.773)	0.00 (−0.03–0.01)	1.00 (0.78–1.38)	0.705 (0.497–0.836)

* Refer to Table 1 for the same annotation. Abbreviations: see Table 1.

The units in Table 3 for certain measurands were incorrectly stated, which needs to be corrected.

**Table 3 diagnostics-16-02446-t003:** Stability test comparing VQ-Tube SST with 5 min and 30 min clotting times.

Measurand	VQ5	VQ30	*p*	Bias (%) (95% CI)	Intercept (95% CI)	Slope (95% CI)	ρ (95% CI)
	Mean/Median						
AST, U/L	21.5 (18.0–25.6)	21.0 (18.0–25.0)	0.09	0.00 (0.00–3.45)	0.00 (0.00–0.00)	1.00 (1.00–1.00)	0.989 (0.980–0.995)
ALT, U/L	14.0 (11.4–21.0)	14.0 (12.0–22.0)	0.72	−0.04 (−2.18–2.09) *	0.00 (−0.72–0.50)	1.00 (1.00–1.00)	0.974 (0.949–0.986)
GGT, U/L	13.5 (12.0–30.1)	13.0 (12.0–30.7)	0.02	0.00 (0.00–0.00)	0.00 (0.00–0.00)	1.00 (1.00–1.00)	0.996 (0.992–0.998)
ALP, U/L	55.0 (47.0–82.0)	55.0 (46.8–82.0)	0.31	0.00 (0.00–0.00)	0.00 (0.00–0.00)	1.00 (1.00–1.00)	0.999 (0.997–0.999)
TP, g/dL	7.2 (6.9–7.4)	7.2 (6.9–7.4)	0.93	0.00 (0.00–0.00)	0.00 (−0.90–0.00)	1.00 (1.00–1.13)	0.985 (0.971–0.992)
Alb, g/dL	4.5 (4.4–4.7)	4.5 (4.4–4.7)	0.08	0.00 (0.00–0.00)	0.00 (0.00–0.00)	1.00 (1.00–1.00)	0.891 (0.798–0.942)
TB, mg/dL	0.62 (0.47–0.69)	0.61 (0.47–0.70)	0.47	0.00 (−1.19–0.00)	0.00 (−0.01–0.02)	1.00 (0.97–1.00)	0.992 (0.985–0.996)
DB, mg/dL	0.12 (0.09–0.14)	0.12 (0.09–0.13)	0.25	0.00 (−5.56–0.00)	0.00 (−0.01–0.01)	1.00 (0.88–1.00)	0.968 (0.939–0.983)
BUN, mg/dL	12.8 (10.4–16.6)	12.9 (10.3–16.6)	0.66	0.00 (0.00–0.64)	0.00 (−0.11–0.16)	1.00 (0.99–1.01)	0.999 (0.997–0.999)
Cr, mg/dL	0.75 (0.66–0.82)	0.74 (0.66–0.82)	0.10	−0.40 (−1.03–0.23) *	0.00 (−0.01–0.04)	1.00 (0.94–1.00)	0.990 (0.980–0.995)
Cholesterol, mg/dL	187.9 ± 35.3 *	188.3 ± 35.2 *	0.24 *	0.22 (−0.13–0.58) *	0.92 (−2.49–4.33) *	1.00 (0.98–1.02) *	0.998 (0.997–0.999) *
LDL, mg/dL	106.6 ± 31.3 *	106.7 ± 31.3 *	0.63	0.00 (−0.68–0.80)	0.02 (−1.61–1.65) *	1.00 (0.99–1.02) *	0.999 (0.998–1.000) *
HDL, mg/dL	63.4 ± 12.8 *	63.7 ± 12.8 *	0.35	0.00 (0.00–1.19)	0.47 (−1.29–2.22) *	1.00 (0.97–1.03) *	0.993 (0.986–0.996) *
TG, mg/dL	102.0 (62.8–158.8)	102.0 (62.4–159.2)	0.06	0.00 (−0.87–0.00)	0.00 (−1.00–0.51)	1.00 (0.99–1.01)	1.000 (1.000–1.000)
Glucose, mg/dL	100.0 (97.0–115.0)	99.5 (93.0–110.0)	<0.0001 *	−3.08 (−3.76–−2.40) *	−3.00 (−5.00–0.86)	1.00 (0.97–1.02)	0.982 (0.965–0.991)
UA, mg/dL	4.60 (3.99–5.29)	4.59 (3.97–5.29)	<0.0001 *	−0.68 (−0.96–−0.39) *	−0.03 (−0.07–0.03)	1.00 (0.99–1.01)	0.996 (0.993–0.998)
LDH, U/L	147.0 (129.4–168.0)	148.5 (137.0–177.2)	<0.0001 *	4.60 (2.71–6.49) *	10.91 (−1.79–29.0)	0.97 (0.84–1.07)	0.934 (0.876–0.966)
CK, U/L	99.5 (76.0–151.7)	97.0 (76.0–150.3)	0.24 *	−0.64 (−1.28–−0.002) *	−1.00 (−3.12–1.02)	1.00 (0.98–1.03)	0.997 (0.995–0.999)
Amylase, U/L	61.0 (44.8–71.2)	60.5 (44.8–71.2)	0.76 *	0.00 (0.00–1.52)	0.00 (−1.18–1.11)	1.00 (0.98–1.03)	0.994 (0.988–0.997)
Lipase, U/L	25.5 (15.4–33.0)	25.5 (15.4–32.0)	0.34	0.00 (0.00–0.00)	0.00 (−0.27–0.00)	1.00 (1.00–1.02)	0.995 (0.990–0.997)
TIBC, μg/dL	337.9 ± 51.5 *	338.1 ± 50.7 *	0.53	0.29 (0.00–0.38)	5.11 (−2.33–12.54) *	0.99 (0.96–1.01) *	0.997 (0.995–0.999) *
Na, mmol/L	138.9 ± 2.2 *	140.0 (138.0–140.0)	0.051	0.00 (0.00–0.71)	0.00 (−27.60–1.00)	1.00 (1.00–1.20)	0.850 (0.728–0.920)
K, mmol/L	4.0 (3.9–4.3)	4.15 ± 0.32 *	0.01 *	0.00 (0.00–2.50)	0.00 (−0.70–0.10)	1.00 (1.00–1.20)	0.945 (0.897–0.971)
Cl, mmol/L	104.0 (103.0–105.0)	104.0 (103.0–105.0)	0.58	0.00 (0.00–0.00)	0.00 (−10.40–0.00)	1.00 (1.00–1.10)	0.884 (0.787–0.939)
Mg, mg/dL	0.9 (0.8–0.9)	0.9 (0.8–0.9)	0.31	0.00 (0.00–0.00)	0.00 (0.00–0.00)	1.00 (1.00–1.00)	0.759 (0.579–0.868)
Ca, mg/dL	9.70 (9.44–9.90)	9.70 (9.50–10.00)	0.01	0.00 (0.00–1.00)	0.00 (0.00–0.10)	1.00 (1.00–1.00)	0.978 (0.957–0.989)
P, mg/dL	3.87 ± 0.44 *	3.88 ± 0.46 *	0.46 *	0.27 (−0.30–0.77)	−0.15 (−0.44–0.14) *	1.04 (0.97–1.11) *	0.993 (0.987–0.996) *
Fe, µg/dL	90.9 ± 36.3 *	91.9 ± 36.0 *	0.001	0.74 (0.00–1.22)	1.77 (0.43–3.12) *	0.99 (0.98–1.00) *	0.998 (0.996–0.999) *
CRP, mg/L	0.60 (0.26–1.01)	0.57 (0.26–1.02)	0.49	0.00 (−1.54–0.00)	0.00 (−0.01–0.002) *	1.00 (0.99–1.02)	0.996 (0.992–0.998)

* Refer to Table 1 for the same annotation. Abbreviations: VQ5, VQ-Tube SST with 5 min clotting time; VQ30, VQ-Tube SST with 30 min clotting time; others in Table 1.

## Missing Citation

In the original publication [1], Bland, J.M.; Altman, D.G. Correlation in restricted ranges of data. *BMJ* **2011**, *342*, d556. was not cited. The citation needs to be inserted between the 5th and 6th paragraphs in the Discussion section as the 25th reference. The correct text appears below.

Although correlation is commonly used for method comparison, it has inherent limitations. It primarily reflects the strength of the linear relationship between two methods and does not necessarily indicate agreement or comparability [17]. Furthermore, a high correlation coefficient does not guarantee the absence of bias [17,25]. Additionally, the restricted range of the measurands also lowers the correlation coefficient [25]. In our study, many measurands with lower correlation coefficients were associated with a narrow distribution range.

## Text Correction

There were errors in the original publication.

(1) In the Results paragraph of the Abstract section, the statement regarding the comparison with the EFLM criteria was missing. The correct text appears below.

**Results**: The V-Tube SST, VQ-Tube SST, and V-Tube K_2_EDTA demonstrated comparable analytical performance to the BD tubes for the majority of measurands. However, glucose, lactate dehydrogenase, mean corpuscular volume, and mean corpuscular hemoglobin concentration indicated clinically significant differences according to the desirable biases referenced from the Desirable Biological Variation Database specifications (Ricos) and the European Federation of Clinical Chemistry and Laboratory Medicine (EFLM).

(2) In the Conclusions paragraph of the Abstract section, the statement missed the results of the EDTA tube comparison. The correct text appears below.

**Conclusions**: These findings suggest that, while the V-Tube SST, VQ-Tube, and V-Tube K_2_EDTA generally serve as alternatives to BD tubes, caution should be taken when interpreting results for specific measurands that demonstrated clinically significant discrepancies.

(3) In the second paragraph of Section 1 (Introduction), the connective phrase needs to be corrected to ensure logical consistency. The correct text appears below.

Serum separator tubes (SSTs) and ethylenediaminetetraacetic acid (EDTA) tubes are widely used in clinical laboratories. SSTs, routinely used in clinical chemistry and immunology examinations, are designed with a clot activator and a gel barrier to promote the efficient separation of serum from cellular components following centrifugation [3]. Conventional SSTs require a 30 min clotting time and 10 min of centrifugation prior to analysis. However, quick-clotting SSTs utilize a thrombin-based clot activator that significantly reduces the clotting time to approximately 5 min [3], which is expected to enhance workflow efficiency without compromising analytical performance.

(4) In the fifth paragraph in Section 1 (Introduction), the EFLM criteria were incorrectly cited as the 2024 criteria. (The actual data retrieval from the EFLM database was performed in July 2025). Additionally, the evaluation of measurement stability was erroneously described as analyte stability. The correct text appears below.

This study aimed to comprehensively evaluate the measurement performance of V-Tube SST, VQ-Tube SST, and V-Tube K_2_EDTA in comparison with the corresponding BD SST and BD K_2_EDTA across a broad panel of chemistry, immunology, and hematology measurands. The clinical significance was assessed according to the desirable bias from the Desirable Biological Variation Database specifications (Ricos) and the European Federation of Clinical Chemistry and Laboratory Medicine (EFLM) [12,13]. In addition, this study assessed the measurement stability in samples collected using the VQ-Tube SST under defined storage conditions.

(5) In the first paragraph of Section 2.3 (Statistical Analysis), the statistical terms were inaccurately described. The correct text appears below.

Normality was assessed using the Shapiro–Wilk test. The error for a single paired measurement was defined as the difference between the candidate tube and reference tube result. For errors showing a parametric distribution, paired *t*-tests were performed; otherwise, the Wilcoxon signed-rank test was applied [1], with statistical significance set at *p* < 0.05. Bias, representing systematic difference, was calculated as the mean (or median) of errors, $\frac{1}{N}\sum_{i=1}^{N}candidate\;\left(i\right)-reference\;(i)$, to minimize the imprecision by averaging [14,15]. Additionally, bias can be represented proportionally as the mean (or median) of proportional errors, $\frac{1}{N}\sum_{i=1}^{N}\frac{candidate\;\left(i\right)-reference\;(i)}{reference\;(i)}$ [15]. Bias was considered statistically significant if its value and 95% confidence interval (CI) did not include zero (line of equality) [14]. Difference plots visualize the bias between two methods, assuming a constant linear bias within a specified interval; the reference tube measurements were plotted on the horizontal axis [14,15]. Clinically significant differences were defined as cases in which the biases and their 95% CI exceeded the allowable limits of desirable biases [16], according to Ricos [12] and EFLM [13], as of July 2025.

(6) In the first and third paragraphs in Section 3 (Results), there were statistical inaccuracies, numerical errors, and grammatical issues. Additionally, the content meant for the 1st paragraph was misplaced in the third paragraph, disrupting the overall flow. To rectify this, the sentences from the original third paragraph need to be moved to the 1st paragraph, the numerical errors corrected, and the third paragraph needs to be deleted. The correct text appears below.

In the comparison of chemistry and immunology measurands between V-Tube SST and BD SST, statistically significant differences were observed for 14 measurands (Cr, HDL, UA, LDH, amylase, K, Ca, P, AFP, CK-MB, fT4, TSH, HBsAg, and HIV Ag/Ab). HDL, amylase, P, and AFP showed positive biases in the V-Tube SST, whereas Cr, UA, LDH, fT4, HBsAg, and HIV Ag/Ab showed negative biases compared to BD SST (Table 1). The biases (%) ranged from −8.86% to 2.49% between V-Tube SST and BD SST. The biases and 95% CIs for all measurands were within the allowable limits defined by Ricos and EFLM. In the comparison between VQ-Tube SST and BD SST, statistically significant differences were identified for 16 measurands (TD, TB, LDL, HDL, TG, glucose, UA, LDH, Na, Mg, P, AFP, CK-MB, fT4, TSH, and HBsAg). HDL, TG, glucose, and AFP showed positive biases in the VQ-Tube SST, whereas UA, LDH, fT4, and HBsAg showed negative biases. The biases ranged from −7.87% to 3.45% between VQ-Tube SST and BD SST. The biases and 95% CIs for all analytes were within the Ricos and EFLM allowable limits, except glucose and LDH. Glucose showed a clinically significant positive bias (3.45%; 95% CI, 2.11–6.67%), and LDH showed a clinically significant negative bias (−7.87%; 95% CI, −10.25–−5.49%) (Figure 1).

(7) In the fourth paragraph of Section 3 (Results), the statistical description is inaccurate, and a sentence was misplaced, disrupting the overall flow. The correct text appears below.

In the stability test of VQ-Tube SST, statistically significant differences were observed in GGT, glucose, UA, LDH, K, Ca, and Fe (Table 3). LDH showed positive biases in the VQ-Tube SST with 30 min clotting time, whereas glucose, UA, and CK showed negative biases. The biases (%) ranged from −3.08% to 4.60%. For almost all measurands, the biases and 95% CIs were within allowable limits, although glucose exceeded the Ricos and EFLM. Glucose showed a clinically significant negative bias (−3.08%; 95% CI, −3.76–−2.40%) (Figure 2). All measurands showed high to very high correlation between VQ-Tube SST with 5 min and 30 min clotting times.

(8) In the fifth paragraph of the Results section, the statistical description is inaccurate, and a sentence was misplaced, disrupting the overall flow. The correct text appears below.

Among hematological measurands, Hct, MCV, MCHC, RDW, and ESR showed statistically significant differences between V-Tube K_2_EDTA and BD K_2_EDTA tubes. Hct, MCV, IRF, and eosinophil showed positive biases in the V-Tube K_2_EDTA, whereas MCHC showed negative biases. (Table 4). The biases (%) ranged from −5.71% to 6.83%. The biases and 95% CIs for all measurands were within the Ricos and EFLM, with the exception of MCV and MCHC. MCV showed a clinically significant positive bias (1.87%; 95% CI, 1.55–2.13%), whereas MCHC showed a clinically significant negative bias (−1.63%; 95% CI, −2.01–−1.26%) (Figure 3).

(9) In the first paragraph in Section 5 (Conclusions), the specific term regarding the measurement examination has been omitted. The correct text appears below.

This study demonstrated that the V-Tube SST, VQ-Tube SST, and V-Tube K_2_EDTA are clinically interchangeable with the BD SST and BD K_2_EDTA for routine chemistry, immunology, and hematology examinations. However, the VQ-Tube SST requires careful consideration when used for glucose and LDH measurements, given the observed clinically significant differences. Although the EDTA concentration in the V-Tube K_2_EDTA complies with established standards, its influence on MCV and MCHC highlights the potential impact of tube composition on hematological parameters. These findings underscore the necessity of tube-specific validation prior to clinical implementation. Further studies involving larger and more diverse subjects are needed to ensure reliable and consistent laboratory results across different blood collection systems.

## References

With the inclusion of “Bland, J.M.; Altman, D.G. Correlation in restricted ranges of data. *BMJ*
**2011**, *342*, d556”, as the 25th reference, the order of some references has been adjusted accordingly.

The authors state that the scientific conclusions are unaffected. This correction was approved by the Academic Editor. The original publication has also been updated.

## Figures and Tables

**Figure 1 diagnostics-16-02446-f001:**
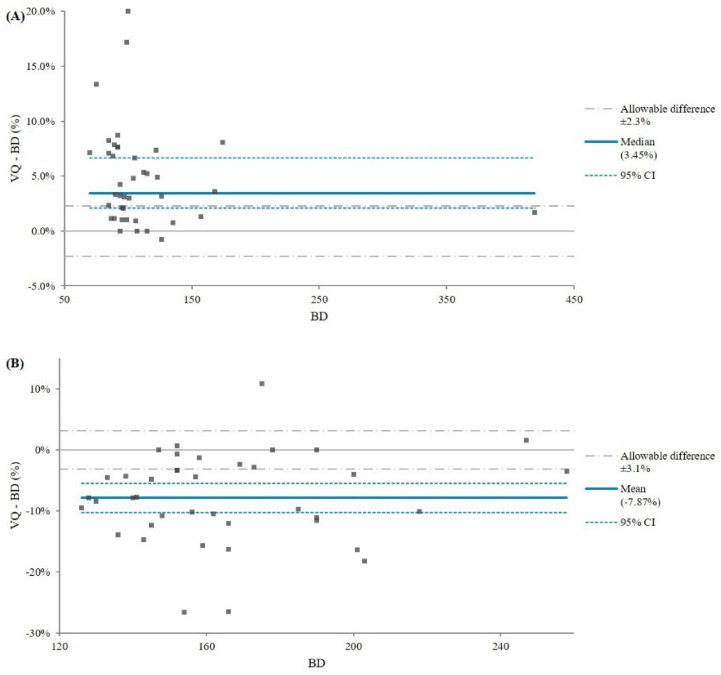
Difference plots for glucose and LDH comparing BD SST and VQ-tube SST. (**A**) Glucose and (**B**) LDH. For glucose, the bias and 95% CI (3.45%, 2.11–6.67%) overlapped with the EFLM desirable bias of 2.3%, but not with the Ricos desirable bias of 1.8%, and for LDH, the bias and 95% CI (−7.87%, −10.25–−5.49%) did not overlap with the EFLM desirable bias of 3.1%.

**Figure 3 diagnostics-16-02446-f003:**
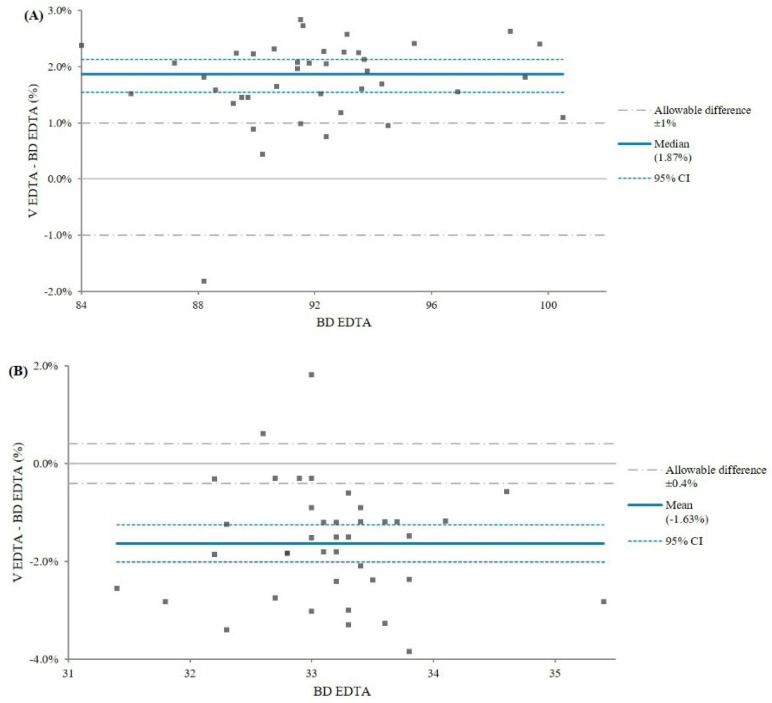
Difference plots comparing V-Tube K_2_EDTA and BD K_2_EDTA for: (**A**) MCV, (**B**) MCHC. For MCV, the bias and 95% CI (1.87%, 1.55–2.13%) did not overlap with the EFLM desirable bias of 1.0%, and for MCHC, the bias and 95% CI (−1.63%, −2.01–−1.26%) did not overlap with the EFLM desirable bias of 0.4%.

## References

[1] Kang T., Yun S.G., Nam M.-H., Cho Y., Nam M. (2025). Comparative Evaluation of Serum Separator V-Tube™, VQ-Tube™, and K_2_EDTA V-Tube™ with Becton Dickinson Tubes for Chemistry, Immunology, and Hematology Examinations. Diagnostics.

